# In Situ Pixel-Scale Magnetic Programming 3-Dimensional Printing for Multimode Soft Miniature Robots with Multifunctions

**DOI:** 10.34133/research.0734

**Published:** 2025-07-22

**Authors:** Song Zhao, Liwen Zhang, Kuntao Tan, Shengbin Zhang, Botao Ma, Xueshan Jing, Xinzhao Zhou, Yan Wang, Huawei Chen

**Affiliations:** ^1^ School of Mechanical Engineering and Automation, Beihang University, Beijing, China.; ^2^ Beijing Advanced Innovation Center for Biomedical Engineering, Beihang University, Beijing, China.

## Abstract

Magnetic microrobots with noncontact and real-time control capabilities have garnered marked attention for targeted drug delivery in narrow, enclosed pathways within the human body. The manufacturing method of these magnetic robots plays a crucial role in determining their functionality. In this study, a photocuring 3-dimensional (3D) printing technique with in situ pixel-scale magnetic programming was developed, enabled by a 3D large-scale uniform magnetic field generator with a high strength of approximately 50 mT. Magnetic particles were rotated and aligned on demand to print intelligent structures with a spatial resolution of 50 μm. A novel key-node splicing magnetization method was introduced to control multicurved deformations in 1D strips and 2D membrane magnetic robots, enabling various modes of locomotion, such as rolling, creeping, swimming, and patch-based drug release. To support additional functions, 3D spatial magnetization was implemented for customized spiral capsule robots, allowing precise multidirectional swimming and multitarget droplet-based drug delivery. These multimode and multifunctional magnetic actuators were validated through in vivo operations in confined environments such as the gastrointestinal tract and bladder.

## Introduction

Gastrointestinal and urinary system diseases are associated with substantial morbidity, mortality, and high healthcare costs [1,2]. Common drug treatments, such as oral medication or intravenous injection, have drawbacks, including low drug concentration at the target site, non-site-specific drug release, and short retention times, leading to reduced treatment efficacy and prolonged healing periods [3,4]. Targeted drug delivery using microrobots has gained widespread attention due to its advantages, such as higher drug concentration, longer retention time, and reduced drug-induced injury [5–13]. However, various anatomical pathways, such as the intestine and bladder, present rugged, meandering, and mucus-covered environments, where existing microrobots with single-mode motion and basic manipulation capabilities struggle to accurately navigate to the targeted regions [14,15]. Therefore, multiple modes of locomotion, diverse functions, and even in situ reshaping capabilities are needed to enable high-precision drug delivery and manipulation in such complex environments.

Magnetic robots, with their advantages of noncontact actuation, deep tissue penetration, and nonradiative operation, are ideal candidates for drug delivery in bodily pathways [16–22]. To enable on-demand deformation of magnetic robots, mold-assisted pre-deforming magnetization methods were initially explored [23–28].

However, these approaches are limited in the complexity of achievable deformations due to the challenges associated with producing and utilizing intricate deformation molds [29–33]. More customizable fabrication methods have emerged, offering real-time control over the remnant magnetization of each robot component. These include nozzle magnetization during direct ink writing, laser-induced local remagnetization, and patterned photocuring under variable magnetic fields [3–39]. For example, Kim et al. [40] employed ink-based 3-dimensional (3D) printing with a ring-shaped permanent magnet to encode magnetization into filament composites with diverse functionalities. Martin et al. [41] integrated stereolithography with soft magnetic colloids to enable real-time particle assembly for tuning material strength. Xu et al. [42] utilized ultraviolet (UV) lithography to encode magnetization into submillimeter-scale planar flexible composites containing permanent magnets. Their work demonstrated the fabrication of millimeter-scale actuators with 3D magnetization arrangements, enabling higher-order and multi-axis deformations. Nonetheless, the lack of a uniform, high-precision 3D magnetic field limits the fabrication of magnetic robots with more sophisticated and accurate magnetization [43–48]. Consequently, a design theory that integrates magnetization patterns with structural configurations for multimode, multifunctional magnetic robots is also needed.

In this study, in situ pixel-scale magnetic programming 3D printing was implemented by integrating a large-scale, uniform, and robust 3D magnetic field generator composed of 2 parallel, rotatable NdFeB permanent magnets. During the magnetic programming process, magnetic particles are rotated on demand and photocured in real time at high angles and fine spatial resolution, enabling in situ pixel-scale magnetization in conjunction with 3D structure formation. To address the complex, rough, and mucus-covered surfaces of human biological pathways, a variety of magnetic robots with distinct shapes and magnetization patterns were designed and fabricated. 1D strips and 2D membrane-type magnetic robots were developed with multicurved deformation capabilities to support varied modes of motion and patch-based drug release, targeting elongated intestinal tracts or liquid–air interface conditions in the bladder. To enable more precise and advanced functions, 3D spiral capsule-shaped magnetic robots were fabricated through 3D spatial magnetization. These robots demonstrated accurate multidirectional swimming and multitarget droplet-based drug release. Finally, in vivo experiments were conducted using these magnetic robots in various anatomical pathways to validate their multimode locomotion and multifunctional manipulation capabilities.

## Results

### Multimode magnetic robots for multifunctional biomedical operations

**Fig. 1.**
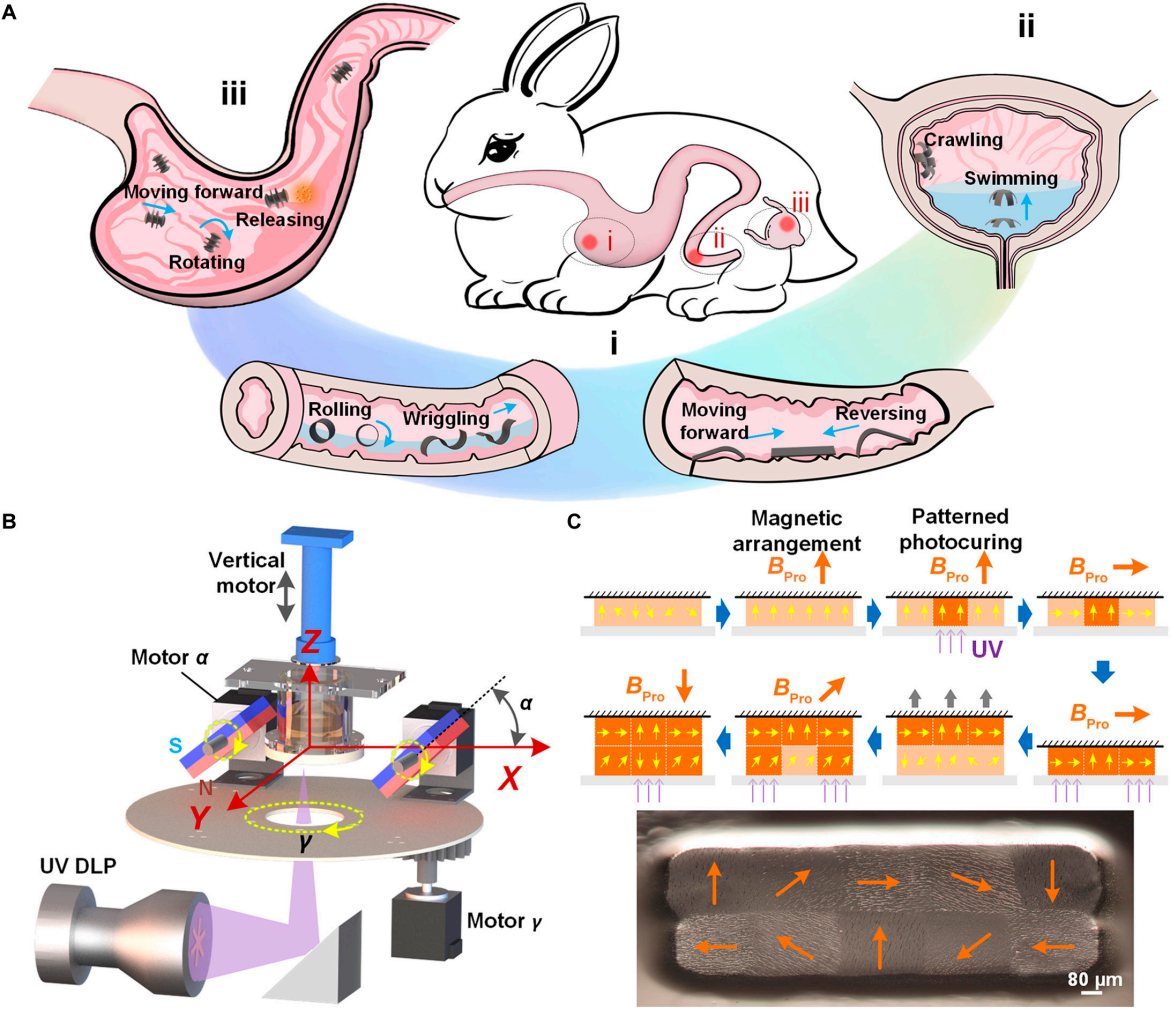
Magnetic robot with complex, multimodal manipulation capabilities for diverse anatomical pathways. (A) Various anatomical pathways—such as the stomach (iii), intestinal tract (i), and bladder (ii)—present complex and markedly divergent environments, requiring medical robots to adopt diverse shapes and deformation modes for multimodal movement and multifunctional operations. (B) The design of an in situ pixel-scale magnetic programming 3D printer integrates a 3D magnetic field generator with a photocuring 3D printer. Rotations *α* and *γ* are applied to 2 parallel permanent magnets to generate a large-scale, uniform 3D magnetic field. (C) Magnetic programming 3D printing process: A programming magnetic field *B*_Pro_ is first applied in a set direction to align magnetic particles within the photocuring resin. A UV mask image is then projected to solidify patterned pixels, thereby fixing the remanent magnetization direction. Different magnetic moment densities within the structure are achieved by altering the *B*_Pro_ direction and photocuring patterns, enabling high-resolution magnetic programming and structural fabrication.

To meet the demands of precision medical interventions within complex biological cavities, magnetic robots must perform various locomotion modes and operations in different regions of the human body—such as omnidirectional rolling and drug release in the stomach, clawing in the long and narrow intestinal tract, and crawling or swimming in the bladder (Fig. 1A). To fabricate magnetic robots with intricate structures and finely tuned functional deformations, a high-precision, in situ pixel-scale magnetic programming 3D printing technique was developed. This method integrates a 3D uniform magnetic field generator with a mask image projection-based photopolymerization 3D printer (Fig. 1B; see also Movie S1 and Table S1). Premagnetized NdFeB microparticles were uniformly mixed into the photocuring resin to serve as the base material for the robots. During the printing process, the programming magnetic field *B*_Pro_ was first adjusted to a specified direction using the generator, aligning the magnetic particles in the precursor resin accordingly. A UV mask image was then projected to cure selected pixels, locking the aligned particle orientation. By repeating this process, the remanent magnetization direction of each pixel could be individually programmed (Fig. 1C, top). After sequential layer-by-layer curing, a 3D structure with pixel-level magnetic programming was obtained (Fig. 1C, bottom). This 3D printing approach combines in situ magnetic programming with fine-scale structural fabrication, offering a powerful platform for creating diverse multimodal and multifunctional magnetic miniature robots.

### In situ pixel-scale magnetic programming 3D printing

**Fig. 2.**
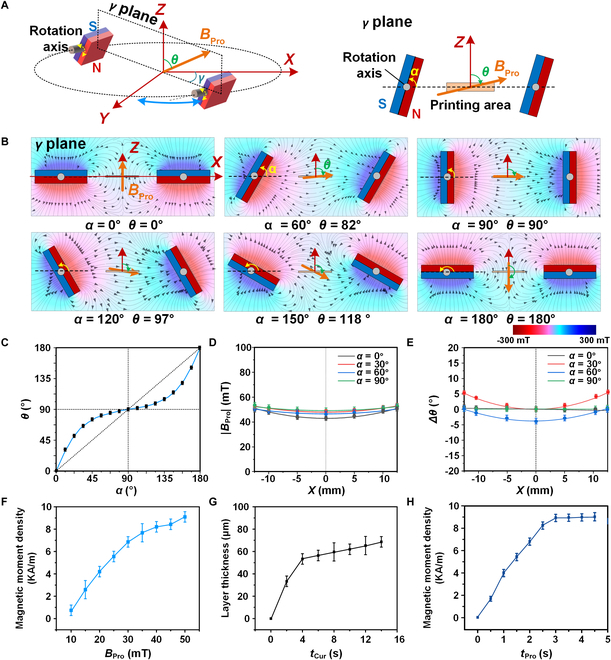
Design and construction of in situ pixel-scale magnetic programming 3D printing platform. (A) The magnetic field generator, with α and γ rotations, generates a large-scale, uniform programming magnetic field *B*_Pro_, denoted as *B*_Pro_(*θ*, *γ*). (B) Simulation of the magnetic field distribution in the generator under different *α* values, demonstrating large-scale coverage and uniformity in both strength and direction. The angle *θ* of *B*_Pro_ counter-rotates in response to changes in magnet angle *α*. (C) Relationship between *α* and *θ*: The desired *θ* of *B*_Pro_ can be achieved by setting the magnets to the corresponding *α* angle. (D and E) As *α* changes from 0 to 90°, the magnetic field intensity |*B*_Pro_| exhibits high uniformity across a 3 cm × 3 cm printing area, with a strength of 50.3 ± 4.5 mT and directional consistency within ±5°. (F) Magnetic moment density of printed samples under *B*_Pro_ of varying magnitudes. (G) Thickness of the photocured layer increases with longer curing time *t*_Cur_. (H) Stronger magnetic moment density is achieved with longer programming time *t*_Pro_, allowing magnetic particles more time to align.

To rotate and align the magnetic particles in the precuring resin, the programming magnetic field *B*_Pro_ must be uniform, strong, and precisely adjustable in 3 dimensions. Two parallel NdFeB cubic permanent magnets are rotated synchronously by stepper motors, with rotation angle *α* (Figs. 1B and 2A, left) [44–47]. As the magnets rotate from *α* = 0° to 360°, the resulting field *B*_Pro_ counter-rotates with angle *θ* from 0° to 360° (Fig. 2A, right, B, and C and Note S1). The entire assembly is mounted on a rotating plate to introduce horizontal rotation angle *γ* (Fig. 1B and Fig. S1A). This configuration enables precise 3D control of *B*_Pro_ expressed as *B*_Pro_(*θ*, *γ*). This 3D programming magnetic field demonstrates excellent strength and uniformity. Within a 3 cm × 3 cm square area, *B*_Pro_ reaches an average field strength of 50.3 ± 4.5 mT and directional uniformity within ±5° in all directions (Fig. 2D and E), which is sufficient for aligning particles during magnetic programming. A magnetic field of approximately 50 mT effectively orients NdFeB particles in the photocurable resin before solidification by patterned UV light. The generator also accommodates the resin tank of a digital light processing (DLP) 3D printer, and the magnetic programming 3D printer platform was constructed by integrating the magnetic field generator with the DLP printer (Fig. S1B). The photocuring and magnetic programming characteristics of the particle-mixed resin were evaluated. (Throughout this study, the percentage of magnetic particles refers to their mass fraction in the resin.) During UV exposure, areas as small as one pixel (50 μm × 50 μm) can be polymerized with high resolution (Fig. S2). When the mass fraction exceeds 20%, UV light transmittance is markedly reduced, resulting in incomplete structures (Fig. S3). To balance performance and printability, a 20% mass fraction was used. The magnetic moment densities of printed samples under different *B*_Pro_ strengths were measured to assess magnetic performance (Fig. 2F). The density increased from 0.8 to 9.1 kA m^−1^ as *B*_Pro_ increased from 10 to 50 mT. These values are used in finite element simulations to predict magnetic deformation behavior under external control fields. The magnetic programming time was set to 10 s to ensure that the particles reached a balanced state, resulting in a stable and uniform magnetization. Because magnetic particles block UV light transmission, longer curing times *t*_Cur_ result in thicker layers—up to ~50 μm (Fig. 2G). Similarly, increasing magnetic programming time *t*_Pro_ enhances the magnetic moment density, reaching up to 8.7 KA/m (Fig. 2H). To optimize robotic magnetic actuation, the programming and photocuring parameters were set to *t*_Pro_ = 4.0 s and *t*_Cur_ = 3.0 s, with a 20% particle ratio. The layer thickness can be controlled between 10 and 50 μm by adjusting the vertical motor step size in the *Z* direction.

For fabricating multimodal and multifunctional magnetic robots, the distribution of remanent magnetization must be coordinated with structural design. Printing data were uploaded to the platform, and the magnetic alignment and pixel-wise photocuring processes were repeated (Fig. S4). The entire system was automated, with a microprogrammed control unit managing the magnetic generator and a computer controlling the UV exposure (Fig. S5). Through accurate alignment of magnetic particles and pixel-level photocuring, a multidirectional magnetic moment density with high resolution and uniformity can be achieved, forming the basis for fabricating complex magnetic robots. Enhancing both the strength and uniformity of the magnetic field is essential for advancing this research.

### Design and fabrication of 1D strip magnetic robots

**Fig. 3.**
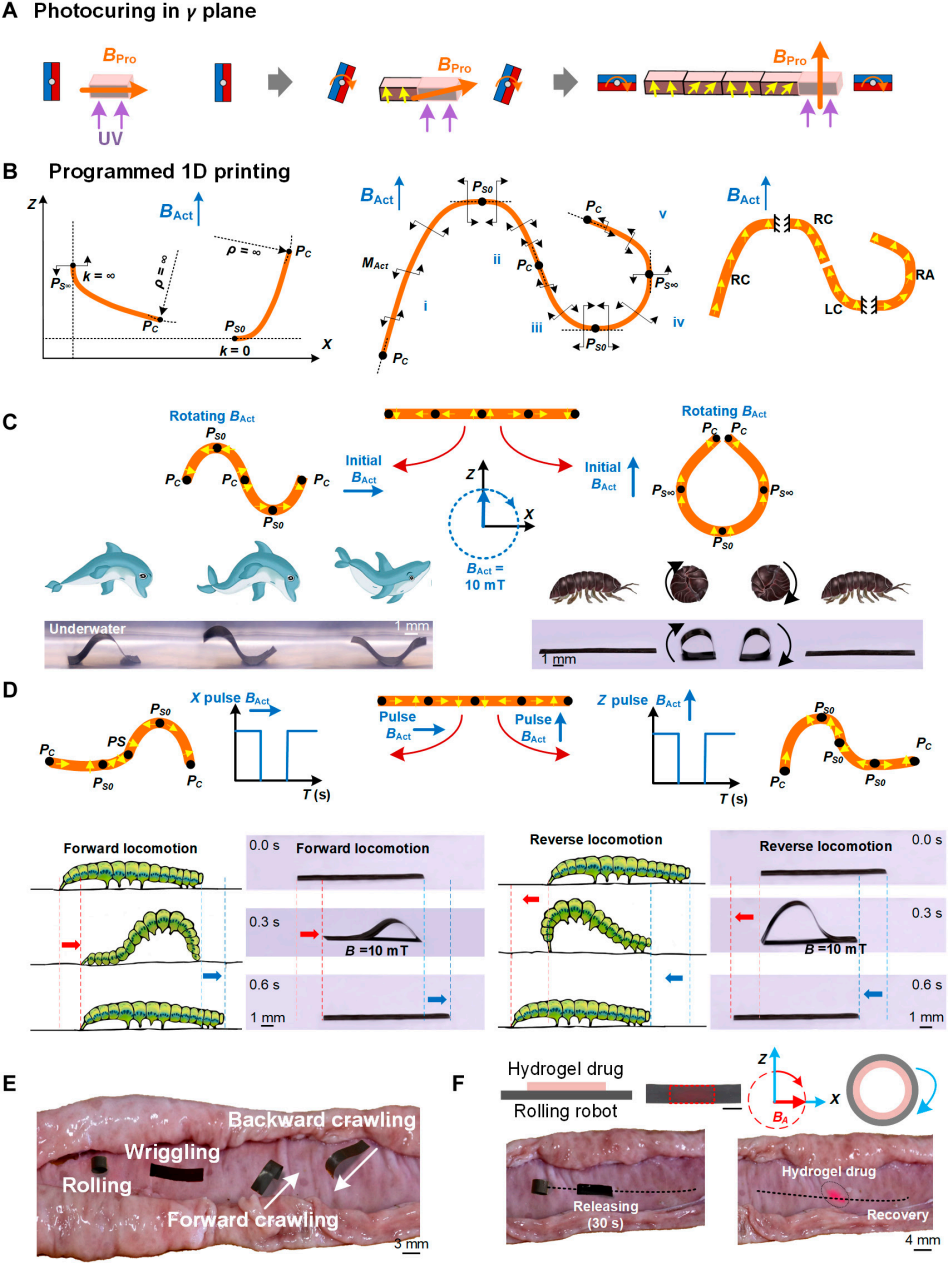
Magnetic programming design and 3D printing of 1D strip robots. (A) A 1D strip magnet exhibiting bending deformation is fabricated by varying *θ* in a fixed *γ* plane during the application of the programming magnetic field *B*_Pro_. After magnetic programming, the required pixels are linearly photocured along the *γ* plane. (B) A key-node splicing magnetization method is proposed based on stress distribution in multicurved deformations. In the *X*–*Z* coordinate system, with *Z* aligned to the actuation magnetic field *B*_Act_, multiple curves are segmented into simple cantilever strips at node points where the slope *k* is ∞ or 0 (denoted as P_S∞_ and P_S0_), or where the curvature *ρ* is 0 (denoted as P_C_). By selecting appropriate magnetizations for each cantilever, desired multicurved deformations can be achieved. (C) Using the key-node splicing method, 1D strip robots are magnetically programmed with consistent magnetization distributions. When *B*_Act_ is rotated initially in the *X* or *Z* direction, the robots exhibit dolphin-like undulatory motion or beetle-like rolling, with speeds of 1 and 0.5 cm/s, respectively. (D) With a specialized magnetization pattern, a caterpillar-like strip robot is fabricated. It moves forward under pulsed *B*_Act_ in the *X* direction and backward under pulsed *B*_Act_ in the *Z* direction. (E) Demonstration of multimode motion of 1D strip robots in an excised pig intestine tract. (F) The beetle-like robot is capable of carrying a hydrogel patch drug internally and releasing it after rolling to the targeted area.

In the long, narrow, and winding intestinal tract, 1D strip robots are well-suited for long-distance, untethered locomotion and multifunctional manipulation. These functions are achieved under a uniform *B*_Act_, through programmable multicurved deformations with varying radii and directions of curvature. To enable this, the magnetic moment density across different parts of the robot must be precisely tailored in various *θ* directions. This is realized by generating *B*_Pro_ (*θ*, 0°), where *γ* is fixed at 0°, and sequentially programming each pixel. The strip magnetic robots are then photocured linearly along the *γ* plane (Fig. 3A and Fig. S6).

To realize 1D strip robots capable of complex, on-demand, multimodal functions, the distribution of their magnetic moment density needs to be accurately designed to perform complex multicurved deformations under uniform *B*_Act_. A novel key-node splicing magnetization method was established based on the stress distribution in multicurved deformations. By establishing *X*–*Z* coordinate with *Z* aligned to *B*_Act_, key nodes appear at points with slope *k* = ∞ or *k* = 0 (i.e., P_S∞_ or P_S0_), or with curvature *ρ* = 0 (P_C_) (Fig. 3B, left and middle). Because the uniform *B*_Act_ only generates torque *M*_Act_ but not translational force, a torque balance is formed between *M*_Act_ and the strip’s elastic recovery torque. Thus, on multicurved strip, P_S∞_ and P_S0_ correspond to points where the elastic torque direction reverses (i.e., where *M*_Act_ direction reverses with *k* = ∞ or 0), whereas P_C_ corresponds to locations where torque *M*_Act_ reduces to zero with *ρ* = 0, such as the strip’s free end. Using these key nodes P_S_ and P_C_, a complex multicurved strip can be decomposed into several simple cantilever segments, each with distinct bending characteristics. This segmentation simplifies the process of designing tailored magnetization profiles (Fig. 3B, right).

Cantilever strips with a fixed and a free end possess magnetic moment density *θ* angling between 0° and ±90° (Fig. S7A). Based on the location of the fixed end and the rotation direction of *θ*, cantilever strips are classified into 4 types: LC, RC, LA, and RA. By defining the rate of increase in *θ* along the cantilever using a factor *Q*, the resulting bending deformation can be more precisely controlled (Fig. S7B and C and Note S2). With different cantilever types, rotation rates *Q*, and variations in the *B*_Act_, diverse bending deformations in various directions and degrees were demonstrated (Fig. S7D). By selecting appropriate combinations from these deformation options, complex multicurved shapes can be reassembled, and the corresponding magnetic moment density distributions can be generated. This key-node splicing magnetization method thus provides the magnetic programming data required for 3D printing. As a result, 1D strip magnetic robots with complex deformations were precisely designed and fabricated to achieve multimodal and multifunctional capabilities. An automatic auxiliary software tool was developed to identify key nodes on arbitrary hand-drawn curves, split them accordingly, and match suitable cantilever deformation patterns to generate the required magnetization distribution data (Fig. S8).

Using this method, several 1D strip magnetic robots with distinct deformation modes were created. Dolphin-like wave and beetle-like rolling motions were achieved on a single strip through a designed magnetization pattern, activated by a rotating *B*_Act_ with different initial directions (Fig. 3C and Movies S2 and S1). A caterpillar-like robot was also developed to produce asymmetric bending deformations. Through repeated deformation and recovery under pulsed *B*_Act_, unbalanced friction on both sides of the robot generated locomotion. Forward and backward asymmetric movements were realized using *X*-direction and *Z*-direction pulsed *B*_Act_, respectively (Fig. 3D, Fig. S9, and Movie S4). These multimodal motions were validated in an excised intestinal tract with a mucus-covered, rugged surface (Fig. 3E and Fig. S3). Furthermore, drug patch delivery was demonstrated using a beetle-like rolling robot with its inner side coated in a hydrogel drug. After rolling to the target location, the robot flattened, pressing the patch onto the intestinal surface for 30 s to form an adhesive bond. The hydrogel drug was then effectively released (Fig. 3F). These 1D strip magnetic robots demonstrate effective multimodal motion and multifunctional performance in long, narrow, and winding intestinal tracts.

### Design and fabrication of 2D membrane magnetic robots

**Fig. 4.**
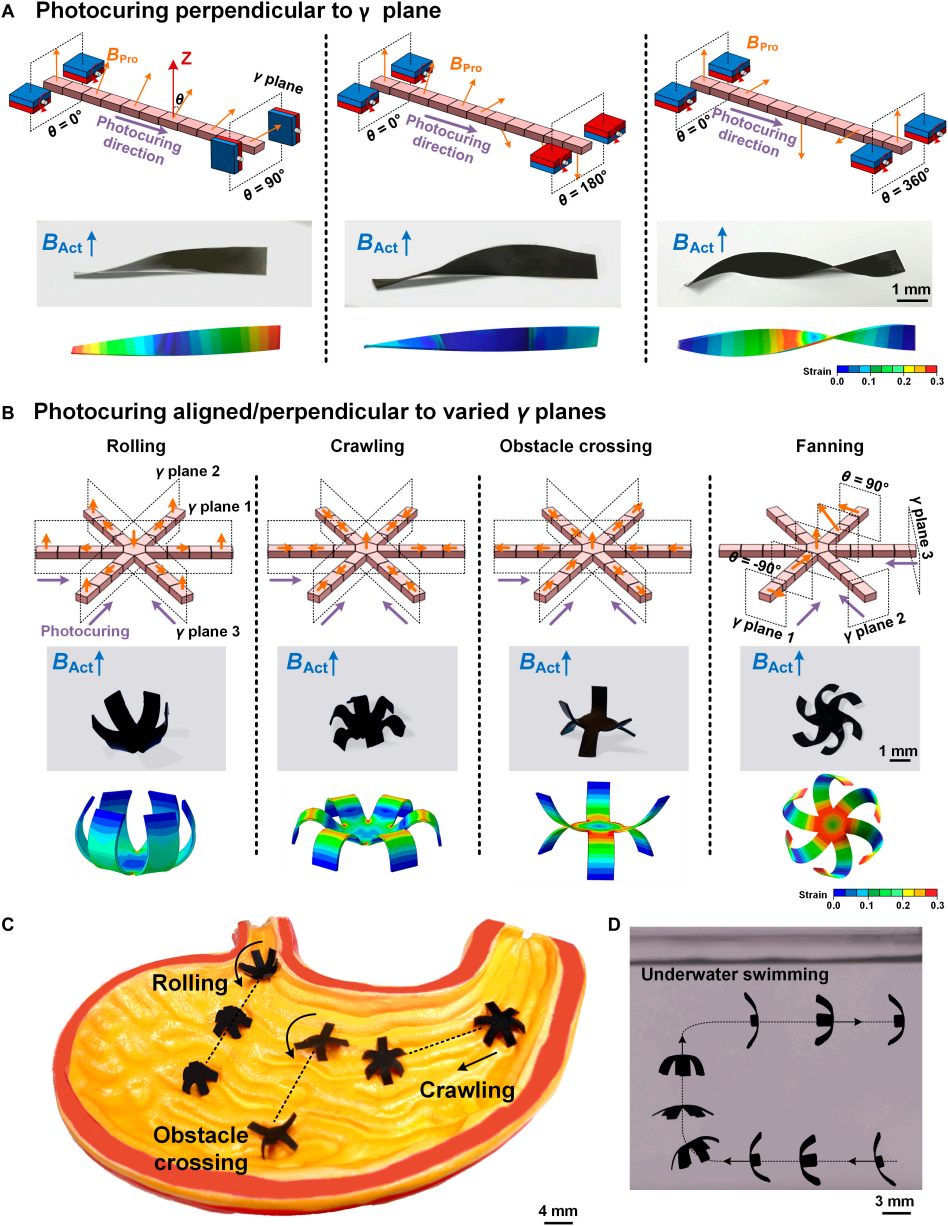
Magnetic programming design and 3D printing of 2D membrane robots. (A) Photocuring in a direction perpendicular to the *γ* plane enables printing of structures with magnetic twisting deformation. Twisting deformations of 90°, 180°, and 360° are demonstrated and validated through magnetic–mechanical coupling simulations. (B) 2D claw membrane magnetic robots capable of rolling, crawling, obstacle crossing, and propelling are designed and fabricated. Claws are printed in 3 intersecting 3 *γ* planes, with photocuring either aligned with or perpendicular to the *γ* planes. (C) Multimodal motions of 2D membrane robots demonstrated in a stomach model. (D) A claw membrane robot swims and steers underwater using pulsed *B*_Act_ at 4 Hz, achieving a speed of 0.6 cm/s.

Large enclosed spaces such as the bladder and stomach, which feature air–liquid mixed environments, require magnetic robots capable of multidirectional movement and various motion patterns. To meet these demands, 2D membrane magnetic robots with protruding claws were developed, enabling more complex deformations. Unlike the in-plane photocuring method used for 1D robots, photocuring pixels perpendicular to the *γ* plane allows the generation of twisting deformations by gradually rotating the *θ* of *B*_Pro_ (Fig. 4A, top). The degree of twisting is precisely controllable by adjusting the range of *θ* (Fig. 4A, bottom). By printing in 3 *γ* planes separated by 60°, claw membrane robots were designed and fabricated to perform a range of multimodal deformations and functions, including rolling, crawling, obstacle crossing, and propelling (Fig. 4B and Fig. S11). These diverse motion capabilities enable the robots to navigate the complex and variable environments of human internal pathways. For instance, rolling, clawing, and obstacle-crossing deformations help the robots traverse ridged surfaces within a stomach model (Fig. 4C, Figs. S12 and S13, and Movie S5). Additionally, by applying a pulsed *B*_Act_, a robot with a crawling deformation was able to swim underwater, mimicking the movement of an octopus (Fig. 4D and Movie S6). Swelling behavior can be redirected simply by changing the direction of *B*_Act_, enabling precise control and enhancing the robots’ potential for targeted drug delivery.

### Design of 3D spiral capsule robot and its multifunctional applications

**Fig. 5.**
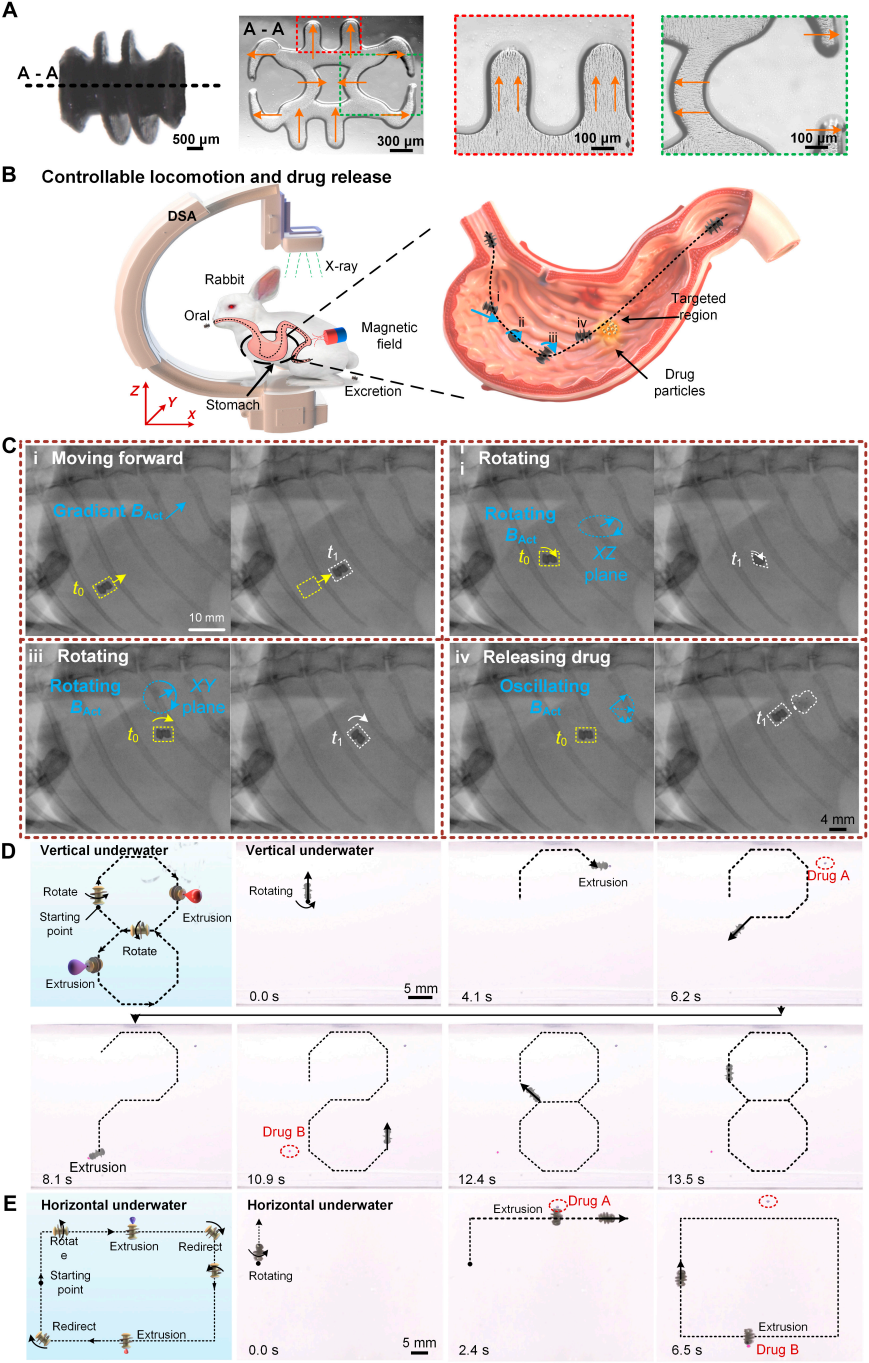
3D spiral capsule magnetic robots with multifunctional control and applications. (A) The 3D spiral capsule magnetic robot is constructed with 2 thin-walled cavities and a surrounding screw-thread line. The thin-walled cavities are oppositely magnetized to enable independent squash deformation for droplet drug release. (B) Schematic of robot actuation (stages i-iii) and drug delivery (stage iv) controlled by an external magnet in a living rabbit’s stomach. Digital subtraction angiography (DSA) monitors real-time status. (Labels i-iv depict the operational sequence; technical implementation via magnetic fields is shown in C.) (C) Various magnetic fields are applied to actuate the spiral capsule robot, enabling spiral propulsion (i), lateral rolling (ii), redirection (iii), and drug release (iv). *t*_0_ and *t*_1_ represent the initial and final states. (D and E) By tuning *B*_Act_ in 3D directions, precise motion is demonstrated through screw-thread propulsion with a pitch of 1.3 mm per rotation. Complex movement paths in “8” and square shapes are shown, along with high-precision droplet drug release.

3D magnetic robots with complex magnetization and structural designs can perform more accurate movements and achieve sophisticated functional combinations. A spiral capsule magnetic robot was developed with 2 back-to-back thin-walled cavities and a surrounding screw-thread structure (Fig. 5A, left). Using a 3D spatial magnetization technique on an in situ pixel-scale programmed printing platform, the 2 cavities were oppositely magnetized to generate independent squash deformations under *B*_Act_ (Fig. 5A, right, and Fig. S14). The thread line was magnetized perpendicular to the magnetization direction of the cavities, allowing separate control of rotational motion via *B*_Act_. With the propulsion provided by the rotating thread line, the robot can move forward or backward, and its displacement can be precisely controlled by adjusting the number of rotations.

To evaluate the robot’s drug delivery performance, an in vivo experiment was conducted in a rabbit. The robot, carrying drug droplets, was administered orally and reached the stomach with the aid of water. Digital subtraction angiography (DSA) was employed to monitor the robot’s status and control in real time, with magnetic actuation applied externally (Fig. 5B and Fig. S15). The time-lapse radiographic video shows the robot tolerating and operating within the viscous and acidic gastric environment (Movie S7). Under different configurations of *B*_Act_, the robot achieved multimodal motion, including spiral propulsion, lateral rolling, and redirection (Fig. 5C, i to iii). Upon reaching the target site, pulsed *B*_Act_ triggered precise drug release via squash deformation (Fig. 5C, iv). After targeted delivery, the robot was fully excreted without causing harm. Biocompatibility tests, detailed in the manuscript, confirmed no marked pathological changes or inflammatory responses (Figs. S16 and S17 and Tables S2 and S3).

Using Helmholtz coils to generate a 3D actuating magnetic field *B*_Act_, the robot demonstrated agile navigation both horizontally and vertically underwater (Fig. 5D and E). By composing *B*_Act_ to counterbalance gravity, vertical spiral-propelling motion was achieved (Note S3). As a result, complex movement paths such as squares, “M” shapes, and “8” figures were precisely executed by continuously adjusting *B*_Act_ (Fig. S18 and Movies S8 and S9). Different drug droplets can be securely stored in the 2 cavities, and directional pulsing of *B*_Act_ can selectively activate each cavity to release its payload at the desired location (Fig. S19). These findings demonstrate the feasibility and precision of the 3D spiral capsule magnetic robot for targeted drug delivery.

## Discussion

To design and produce multimode complex magnetic robots, we developed an in situ pixel-scale magnetic programming 3D printing approach. During magnetic programming printing, magnetic particles are on-demand rotated and arranged to print smart structure. A novel key-node splicing magnetizing method was introduced to control multicurved deformations in 1D strip and 2D membrane magnetic robots, enabling multimode motions. Additionally, a 3D spiral capsule robot with integrated functions was designed and fabricated using 3D spatial magnetization, allowing high-precision, multidirectional motion and multitarget droplet drug release. This method, in contrast to traditional fabrication techniques such as template-assisted molding and lithography, integrates voxel-level geometry and magnetization into a single, automated platform, substantially reducing manual labor and enhancing fabrication efficiency, particularly for complex 3D magnetically responsive robots.

The current study advances the fabrication of magnetic soft robots; however, certain limitations remain that must be addressed to fully realize the potential of this approach. One of the challenges we face is the scalability of the fabrication process, especially when printing larger and more intricate 3D designs. The size of the printed robots is constrained by the printable area, which limits the dimensions of the structures that can be fabricated. Additionally, the magnetic particles used in the photopolymerization process affect the curing properties, meaning that the volume fraction of magnetic particles must be carefully controlled. As a result, the magnetic moment density of the printed magnetic robots is lower compared to those fabricated using traditional molding techniques.

In future work, we plan to extend our work in several directions. First, we aim to refine the precision of the fabricated magnetic profiles and structures to enable more coordinated robot control, including achieving higher magnetization densities and more complex, multimaterial configurations. Additionally, optimizing biocompatibility for clinical applications will be a priority, as we strive to ensure the safety and effectiveness of these robots in medical environments. We will also explore ways to enhance the scalability of our fabrication methods to allow for the manufacturing of larger, more intricate robots with improved performance characteristics. We believe that addressing these limitations and pursuing the suggested future directions will substantially advance the field and provide a strong foundation for subsequent innovations.

## Materials and Methods

### Magnetic photocuring resin material

For the flexible robot, permanent magnetic particles (D50 = 5 μm, MQFP-15-7, NdFeB, Magnequench [Tianjin] Co. Ltd., Tianjin, China) were first magnetized under a strong uniform magnetic field (2.0 T) generated by an impulse magnetizer (2 Tesla Pulse Magnetiser, Magnetic Measurements Ltd., Lancashire) to achieve saturation remanence. To improve particle dispersion and prevent magnetic agglomeration, 5 wt % oleic acid was first added to the magnetized NdFeB particles and mixed thoroughly. Oleic acid acts as a surfactant by adsorbing onto the particle surfaces, thereby reducing interparticle magnetic attraction. The particles were then mixed with a flexible UV resin (Elastic 50 A, FormLabs Inc., MA, USA, or GC3D-EBE Flexible Beige, Smart Your Life, Taiwan). After mixing, the composite was mechanically stirred for 20 min, followed by ultrasonication for 5 min to ensure uniform dispersion of the particles within the resin.

### In situ pixel-scale magnetic programming 3D printer and printing process

The in situ pixel-scale magnetic programming 3D printer consists of 3 functional parts: a 3D large-scale uniform magnetic field generator, a DLP 3D printer, and a programming and printing control unit (Fig. S1). The 3D magnetic field generator comprises 2 parallelly placed strong NdFeB permanent magnets (N35) in size of 4 cm × 4 cm × 2 cm and central magnetic remnant of 350 mT. Two types of rotating movements are designed, i.e., 2 magnets synchronously rotated by 2 stepping motors to create a rotating angle *α*, and then mounted to a horizontal rotating plate around *Z* axis to create rotating angle *γ.* Programming magnetic field magnitude and direction were continuously monitored by using a 3D magnetic Hall effect sensor (TLE493D-W2B6, Infineon Technologies, Munich, Germany). The probe of the Hall element is placed at the designated position, which can display the real-time magnetic field strength and direction of the point.

The resin storage tank of DLP 3D printer is placed between 2 parallel magnets. The photocuring images are produced by a UV DLP source (DLP Light Crafter 4500, Texas Instruments Inc., TX, USA), reflected by a prism, and projected to the bottom of the resin storage tank to photo cure layers of structure on building plate. A vertical linear motor drives the support platform along the *Z* axis for layer-by-layer stacking. Programming and printing control unit consists of an Arduino-based microcontroller to run the 3D magnetic field generator and a computer to project the photocuring images to DLP (Fig. S1). The photocuring images and printing parameters consisting of the exposure and magnetization time and single-layer thickness are loaded into the control unit.

For the printing process, the container filled with magnetic resin is placed above the prism mirror in the 3D printer. The mixed magnetic particles are oriented by *B*_Pro_, which are then exposed by the patterning UV light to cure certain pixels and fix their remanence. During the magnetic printing, *B*_Pro_ is firstly tuned into setting direction by the generator, and the NdFeB microparticles in precuring resin start to rotate and align to the magnetic field. By repeating the magnetic programming and photocuring steps, multilayer with on-demanded magnetization can be achieved. The exposure duration depends on the desired layer thickness and magnetic particle concentration ratio. The printed magnetic robots on build plate are taken out after finish and then washed by ethyl alcohol (Macklin, 99.7%, E809061) to remove uncured resins. To achieve the best performance for robot’s magnetic actuation and soft deformation, the programming and photocuring parameters are set as *t*_Pro_ = 4.0 s and *t*_Cur_ = 3.0 s, and the irradiation power is 5,500 mW. When printing some slender thin-walled tubular structures, in order to ensure that the tubular structures do not collapse or deform during the printing process, support structures made of the same material will be added during the printing process (Note S4). By combining laser cutting with manual trimming using slender precision scissors, the bracket can be removed to minimize potential damage to soft structures.

### Characterization of magnetic programming and material properties

A scanning electron microscope (TESCAN) operated at 5 kV was used to examine the microstructures of the magnetic robots. A vibrating sample magnetometer (VSM; Lake Shore 7410) was used to measure the magnetic moment density of the printed samples for finite element simulations. The magnetic moment densities of the saturated magnetized samples were measured to assess the alignment quality of the magnetic particles. Samples were rectangular (2 × 1 × 1 mm^3^), magnetized along their thickness, and their magnetic moment density was calculated by dividing the remanent magnetization by volume. The magnetic hysteresis loop of the layered film was also measured using the same VSM within an external magnetic field range of −2 to +2 T.

### Actuating magnetic fields for robot movement and manipulation

All actuating magnetic fields used in the experiments were uniform, with a strength of approximately 10 mT, generated by a 3-axis Helmholtz coil (PaiSheng, Hunan). The dolphin-like robot swam in water under a rotating *B*_Act_, initially oriented horizontally, at a speed of 1 cm/s (Fig. 3C, left). The beetle-like robot rolled under a rotating *B*_Act_ with an initial vertical direction at 0.5 cm/s (Fig. 3C, right). The caterpillar-like robot crawled forward under a pulsed *B*_Act_ in the *Z* direction at 1 Hz (Fig. 3D) and crawled backward under a pulsed *B*_Act_ in the *X* direction.

The 2D membrane robots, capable of rolling, crossing obstacles, and crawling on a stomach model, were actuated using a 4 cm × 4 cm × 4 cm N35 permanent magnet with a central remanent magnetic field of 380 mT (Fig. 4C). The 2D robots also swam at 0.6 cm/s under control of the 3-axis Helmholtz coil with magnetic strength of ~30 mT and frequency of 4 Hz.

The 3D spiral capsule-shaped magnetic robot operated in water using the 3-axis Helmholtz coil, achieving a swimming distance of 1.3 mm per rotation (Fig. 5D and E). The speed V was calculated as:

$$V=\frac{D}{t}$$ (1)

where *D* and *t* denote the distance and time, respectively. The composition of the 3D actuating magnetic field in arbitrary directions is provided in Note S3. The same 3D robot, when tested in a rabbit’s stomach, was actuated using a permanent magnet with a field strength of approximately 120 mT.

### Hydrogel-based drug delivery in Fig. 3

The patch physical cross-linked gelatin–chitosan hydrogel (15% w/v gelatin, 5% w/v chitosan, 0.05% w/v eosin Y, Macklin) was prepared as follows: 0.33 g of chitosan dissolved in 5 ml of 1% acetic (Macklin) acid for 60 min formed a clear solution, while 1 g of gelatin dissolved in 1.67 ml of deionized water at 60 °C. The 2 solutions were mixed at 50 to 60 °C, combined with pre-dissolved eosin Y, stirred uniformly, and degassed (1,000 rpm, 5 min). The mixture was poured into sterile, petroleum jelly-coated molds and cooled at 4 °C for 4 h to form cross-links via gelatin–chitosan entanglement. The resulting pink hydrogels were detached from the molds and equilibrated in sterile phosphate-buffered saline (PBS; pH 7.4) for 10 min to neutralize residual acetic acid. The hydrogel’s adhesion stems from synergistic hydrogen bonding (via gelatin’s polar groups) and electrostatic interactions (via protonated chitosan amino groups), enabling robust bonding with both biological tissues and the 1D rolling magnetic robot for stable mechanical integration.

### Drug release mechanism in Fig. 5

In our system (Fig. 5), the deformation and drug release of the magnetic robot are achieved through the coordinated application of 2 magnetic fields: a rotating magnetic field for driving helical motion and a gradient magnetic field for cargo release. The rotating magnetic field is generated by a Helmholtz coil, which is used to drive the robot toward the target position. The gradient magnetic field is generated by a miniature magnet (diameter 3 mm, height 2 mm). The miniature magnet is fixed on a glass rod with a diameter of 4 mm and placed 1 cm away from the release point (the magnetic field generated by the magnet is close to zero at 1 cm). When the magnetic robot reaches the release point, the rotating magnetic field is paused, and the robot continues its previous motion due to inertia. During this brief pause, the miniature magnet rapidly approaches, and the local gradient magnetic field generated by the small magnet causes deformation of the robots’ thin-walled compartment through the combined effect of magnetic force and mechanical interaction with adjacent plastic sidewalls or substrates, leading to the extrusion and release of the encapsulated cargo (originally simulated with colored silicone oil; in this case, water-soluble dye is used to better demonstrate the effect). Afterward, the magnet returns to its original position, and the rotating magnetic field is re-applied, causing the magnetic robot to continue toward the next release point. The same operation is applied to the second release point. It is important to note the timing of the magnet’s proximity to the magnetic robot. When the magnet approaches the robot and acts directly on the thin-walled compartment, the robot may flip (Fig. S19B), which could affect subsequent movement. However, when the magnet approaches the robot at a position between the compartment and the helical structure, the deformation occurs (Fig. S19A). At this point, the compartment reaches a force equilibrium under the influence of the magnetic field, and it is compressed, releasing the drug.

In the in vivo experiments conducted on rabbits, considering the complex physiological environment within the body, a different approach was adopted. A larger magnet (diameter 80 mm, height 30 mm) was used to create an oscillating magnetic field on the rabbit’s abdomen, ensuring that the robot could effectively release the drug within the gradient magnetic field.

### In vivo animal experiments with 1D strip and 3D spiral capsule robots

The 1D strip beetle-like robot shown in Fig. 3F was coated on one side with a gelatin hydrogel drug and actuated in a rolling mode by the *B*_Act_, with the drug carried inside. These robots were tested by rolling through freshly excised pig intestines. The 3D spiral capsule robot was coated with a polydimethylsiloxane (PDMS) layer to reduce the biocytotoxicity of NdFeB and then administered orally to an adult New Zealand rabbit that had been food-restricted for 24 h prior to the experiment. The movement of the robot inside the rabbit was monitored in real time using DSA (Pilot3000, WeMed Medical Equipment Co. Ltd.) (Fig. 5C). After successful drug delivery, the robot was naturally expelled from the rabbit’s body via feces 3 d post-experiment. Ethical approval for the animal experiments was obtained from the Institutional Animal Care and Use Committee (IACUC-D2024004).

### In vivo histological evaluation

The histopathological assessment was performed to evaluate the tissue response after oral administration and subsequent excretion of PDMS-coated magnetic robots in Sprague–Dawley rats, following ISO 10993-1 and ISO 10993-6 guidelines. After fasting for 12 h, rats received a single oral dose of the test material mixed with a standard diet via oral gavage. The animals were euthanized at 7 and 14 d post-administration, and tissues (including the gastrointestinal tract) were collected, fixed in formalin, and processed for paraffin embedding. Tissue sections were stained with hematoxylin and eosin for morphological analysis.

#### Qualitative analysis

##### PDMS-coated magnetic robots

The intestinal structure remained intact, with uniform staining of both the mucosal and muscular layers. Epithelial cells appeared full, round, and densely packed. The crypt architecture was well-preserved without abnormalities, and the submucosal layer showed no marked signs of inflammation.

##### Non-PDMS-coated magnetic robots

Although the overall intestinal architecture was preserved, localized thickening of the intestinal wall was observed (indicated by blue arrows). The crypt structure remained intact with no overt abnormalities, but the submucosal layer exhibited infiltration of macrophages and lymphocytes (black arrows), indicating an inflammatory response. No other pathological changes were noted.

#### Quantitative analysis

##### Non-PDMS-coated group

Histological scores of 4.2 ± 0.8, indicating marked tissue damage and granuloma formation.

##### PDMS-coated group

Histological scores of 1.3 ± 0.4, with minimal tissue damage and mild macrophage infiltration. This analysis shows that the PDMS coating markedly reduces toxicity and tissue damage, demonstrating its biocompatibility for applications involving oral ingestion and excretion.

### In vitro cytotoxicity assay 

The cytotoxicity assessment was conducted using an in vitro cell contact method to evaluate material toxicity, in accordance with ISO 10993-17 guidelines for methylthiazolyldiphenyl-tetrazolium bromide (MTT) assays. L929 fibroblasts were seeded into 96-well plates and cultured for 24 h (one doubling cycle) until reaching 80% confluency. Subsequently, cells were exposed to test material extracts (100% concentration) and control groups (negative/positive controls) for 24 h. Post-exposure, cell morphology was examined via phase-contrast microscopy, and formazan production was quantified spectrophotometrically to calculate relative cell viability against blank controls.

Key results: 1. Negative/positive control validation. Both controls exhibited expected outcomes, confirming assay validity. 2. Qualitative analysis. Non-PDMS-coated magnetic robot: Cytotoxicity grade 4 (severe morphological alterations). PDMS-coated counterparts: Cytotoxicity grade 0 (no observable abnormalities). 3. Quantitative analysis. Non-PDMS group: Cell viability = 14.2% (<70% threshold, indicating marked toxicity). PDMS-coated group: Cell viability = 79.7% (>70%, demonstrating biocompatibility).

### Simulation of 3D large-scale uniform magnetic field generator and magnetic actuated deformation

In the finite-element analysis, the commercial software COMSOL was used to simulate the variation of magnetic field angles under different magnet orientations in the magnetic field generator (Note S5). The robot deformation in response to the actuation magnetic fields was simulated by a user-defined element subroutine in ABAQUS (Note S6).

## Data Availability

All data needed to evaluate the conclusions in the paper are present in the paper and/or the Supplementary Materials. Additional data related to this paper may be requested from the authors.

## Supplementary Materials

Supplementary 1 Note S1 to S6 Figs. S1 to S26 Tables S1 to S3 Movies S1 to S11
