# Supplementary material for: In Situ Pixel-Scale Magnetic Programming 3-Dimensional Printing for Multimode Soft Miniature Robots with Multifunctions
Source: Research (Wash D C). 2025 Jul 22;8:0734. doi: 10.34133/research.0734 (PMC12282394; doi:10.34133/research.0734)
Supplement: Supplementary 1 — Note S1 to S6 Figs. S1 to S26 Tables S1 to S3 Movies S1 to S11 [file research.0734.f1.zip › Revised Supplementary Materials(clean version).docx]

**Supplementary Information for**

***In situ* pixel-scale magnetic programming 3D printing for multi-mode soft miniature robots with multi-functions**

Song Zhao^1#^, Liwen Zhang^1#^, Kuntao Tan^1^, Shengbin Zhang, Botao Ma, Xueshan Jing^1^, Yan Wang^1^, Xinzhao Zhou^1^, Huawei Chen^1,2*^

^1^ School of Mechanical Engineering and Automation, Beihang University, Beijing, China.

^2^ Beijing Advanced Innovation Center for Biomedical Engineering, Beihang University, Beijing, China.

^#^Authors equally contributed to the work.

^*^Email: [chenhw75@buaa.edu.cn](mailto:chenhw75@buaa.edu.cn)

**The PDF file includes:**

Supplementary Note 1 to Note 6

Supplementary Figures 1 to 26

Supplementary Table 1 to 3

Legends for Videos 1 to 11

Supplementary References

**Other Supplementary Material for this manuscript includes the following:**

Supplementary Videos 1 to 11

**Supplementary Note 1. Derivation of analytical formula for three-dimensional magnetic field of shaped permanent magnets**

A rectangular permanent magnet with dimensions *a* × *b* × *h* (mm)^3^, positioned in the Cartesian coordinate system as shown in Supplementary Fig. 20, is uniformly and fully magnetized in the z-direction and saturated, then its internal magnetization vector *M* can be regarded as a constant vector. From the assumption of the amperometric molecular loop, the magnetic field at any point in the external space of the magnet is excited by the closed current loop ABCDA on the side surface of the permanent magnet[1,2]. Here, take the coordinates of the source point of the magnetic field of the permanent magnet as (*x_0_*, *y_0_*, *z_0_*), and the coordinates of the field point *P* as (*x*, *y*, *z*). An analytical expression for the three-dimensional magnetic induction strength in the external space of a permanent magnet saturated with magnetization in the z-direction can be expressed as

$$B_{x}=\frac{K}{2}\left[ -\Gamma\left( a-x,b-y,z \right)-\Gamma\left( a-x,y,z \right)+\Gamma\left( x,b-y,z \right)+\Gamma\left( x,y,z \right) \right]$$

$$B_{y}=\frac{K}{2}\left[ -\Gamma\left( b-y,a-x,z \right)-\Gamma\left( b-y,x,z \right)+\Gamma\left( y,a-x,z \right)+\Gamma\left( y,z,x \right) \right]$$

$$B_{z}=K\left[ -\Psi\left( b-y,a-x,z \right)-\Psi\left( b-y,x,z \right)-\Psi\left( a-x,b-y,z \right)-\Psi\left( x,b-y,z \right)-\Psi\left( b-y,x,z \right)-\Psi\left( y,x,z \right)-\Psi\left( a-x,y,z \right)-\Psi\left( x,y,z \right) \right]$$

$$\begin{aligned} \#\left( S1 \right) \end{aligned}$$

where the expressions of the auxiliary functions Г and Ψ are respectively

$$\Gamma\left( \gamma_{1},\gamma_{2},\gamma_{3} \right)=ln\frac{\sqrt{{\gamma_{1}}^{2}+{\gamma_{2}}^{2}+\left( \gamma_{3}-z_{0} \right)^{2}-\gamma_{2}}}{\sqrt{{\gamma_{1}}^{2}+{\gamma_{2}}^{2}+\left( \gamma_{3}-z_{0} \right)^{2}-\gamma_{2}}}|_{z_{0}=0}^{Z_{0}=h}$$

$$\Psi\left( \psi_{1},\psi_{2},\psi_{3} \right)=arctg\left[ \frac{\psi_{1}}{\psi_{2}}\frac{\left( \psi_{3}-z_{0} \right)}{\sqrt{{\psi_{1}}^{2}+{\psi_{2}}^{2}+\left( \psi_{3}-z_{0} \right)^{2}}} \right]|_{z_{0}=0}^{Z_{0}=h}$$

$$\begin{aligned} \#\left( S2 \right) \end{aligned}$$

and

$$K=\frac{\mu_{0}J_{s}}{4\pi}$$

where the magnetizing current density of the inner surface of the loop is *J_s_*, unit: A/m, and *μ_0_* is magnetic permittivity.

When two magnets separated by *d* and parallelly rotated with the same *α*, as shown in Supplementary Fig. 21 and 22, the coordinate system of magnet No. 1 is still used as the reference coordinate system with

$x_{1}=x\cos\beta+z\sin\beta$, $y_{1}=y$, and $z_{1}=z\cos\beta-x\sin\beta$

The transformation formula for the coordinate system of magnet No. 2 is as follows:

$x_{2}=x\cos\beta+z\sin\beta+d\sin\beta$, $y_{2}=y$, and $z_{2}=z\cos\beta-x\sin\beta-d\cos\beta$

Based on Eq. S1, an analytical expression for the magnetic field between two parallel placed magnets is given by:

$$B_{x}=\frac{k}{2}\left[ -\Gamma\left( a-x_{1},b-y,z_{1} \right)-\Gamma\left( a-x_{1},y,z_{1} \right)+\Gamma\left( x_{1},b-y,z_{1} \right)+\Gamma\left( x_{1},y,z_{1} \right) \right]+\frac{k}{2}\left[ -\Gamma\left( a-x_{2},b-y,z_{2} \right)-\Gamma\left( a-x_{2},y,z_{2} \right)+\Gamma\left( x_{2},b-y,z_{2} \right)+\Gamma\left( x_{2},y,z_{2} \right) \right]$$

$$B_{y}=\frac{K}{2}\left[ -\Gamma\left( b-y,a-x_{1},z_{1} \right)-\Gamma\left( b-y,x_{1},z_{1} \right)+\Gamma\left( y,a-x_{1},z_{1} \right)+\Gamma\left( y,z_{1},x_{1} \right) \right]+\frac{K}{2}\left[ -\Gamma\left( b-y,a-x_{2},z_{2} \right)-\Gamma\left( b-y,x_{2},z_{2} \right)+\Gamma\left( y,a-x_{2},z_{2} \right)+\Gamma\left( y,z_{2},x_{2} \right) \right]$$

$$B_{z}=K\left[ -\Psi\left( b-y,a-x_{1},z_{1} \right)-\Psi\left( b-y,x_{1},z_{1} \right)-\Psi\left( a-x_{1},b-y,z_{1} \right)-\Psi\left( x_{1},b-y,z_{1} \right)-\Psi\left( b-y,x_{1},z_{1} \right)-\Psi\left( y,x_{1},z_{1} \right)-\Psi\left( a-x_{1},y,z_{1} \right)-\Psi\left( x_{1},y,z_{1} \right) \right]+K\left[ -\Psi\left( b-y,a-x_{2},z_{2} \right)-\Psi\left( b-y,x_{2},z_{2} \right)-\Psi\left( a-x_{2},b-y,z_{2} \right)-\Psi\left( x_{2},b-y,z_{2} \right)-\Psi\left( b-y,x_{2},z_{2} \right)-\Psi\left( y,x_{2},z_{2} \right)-\Psi\left( a-x_{2},y,z_{2} \right)-\Psi\left( x_{2},y,z_{2} \right) \right]$$

$$\begin{aligned} \#\left( S3 \right) \end{aligned}$$

This numerical calculation helps the design of 3D large-scale uniform magnetic field generator constructed with two parallel rotating magnets.

**Supplementary Note 2. *θ*'s rotating rate Q in cantilever strip**

As shown in Supplementary Fig. 7a, on cantilever strip the magnetic field direction at position *l* is defined as angle *θ_l_*. Each cantilever strip possesses remnant magnetization with *θ* ranging from 0° to 90°. Define *θ_l_* as

$$\begin{aligned} \theta_{l}=\left( \frac{l}{L} \right)^{Q}\cdot\frac{\pi}{2}\#\left( S4 \right) \end{aligned}$$

where *L* is the strip’s total length and *Q* is the prescribed adjusting coefficients, $Q\in(0,+\infty)$ (Supplementary Fig. 7b). A higher *Q* value means a faster change in remnant magnetization from 0° to 90°. By applying a vertically upward magnetic field, different *Q* corresponds to different degrees of deformation (Supplementary Fig. 7c). For any given multi-curves, it can be split by key-nodes splicing magnetizing method, into various simple cantilever strip. By choosing the demand deformation from Supplementary Fig. 7d, the multi-curves’ magnetization data is achieved for further magnetic programming printing.

**Supplementary Note 3. 3D actuating magnetic field generated by three-axis Helmholtz coils**

The actuating magnetic field *B_Act_* with strength *B* and direction of *α* and *θ*. The magnetic field’s components in x, y and z directions need to meet the following relation,

$$\begin{aligned} \left\{ \begin{matrix} {B_{x}}^{2}+{B_{y}}^{2}+{B_{z}}^{2}=0 \\ B_{x}\sin\theta\cos\alpha+B_{y}\cos\theta\sin\alpha+B_{z}\sin\alpha=0 \end{matrix} \right.\#\left( S5 \right) \end{aligned}$$

Using time t as a time variable, it can be parameterized as:

$$\begin{aligned} \left\{ \begin{matrix} B_{x}=-B\left( \cos\theta\cos t+\sin\theta\sin\alpha\sin t \right) \\ B_{y}=B\left( \sin\theta\cos t-\cos\theta\sin\alpha\sin t \right) \\ B_{z}=B\cos\alpha\sin t \end{matrix} \right.\#\left( S6 \right) \end{aligned}$$

By generating the magnetic field from each coil of the three-axis Helmholtz coils, the required *B_Act_* can be achieved (Supplementary Fig. 23).

**Supplementary Note 4. 3D-printed thin-walled structures**

After completing the *in situ* magnetic programming printing process, two types of thin-walled soft robots were successfully manufactured. In order to ensure that the tubular structure does not collapse or deform during the printing process, a support structure made of the same material was added during the printing process (Supplementary Fig.24a). The supports were removed through a combination of laser cutting and manual trimming using fine-tipped scissors to minimize potential damage to the soft structures. The first prototype, an intestine-inspired peristaltic soft robot, consisted of 200 printed layers with a layer thickness of 50 μm. The total fabrication process, including both printing and support removal, required approximately 1.5 hours. The second prototype, a wave-like peristaltic pump robot, was also composed of 200 layers at 50 μm thickness per layer, with the combined printing and support removal process completed within approximately 2 hours.

The magnetic moment distribution of the intestine-inspired peristaltic robot is presented in Supplementary Fig.26a, with each cross-sectional layer exhibiting an identical total magnetic moment density. When actuated by a permanent magnet generating a magnetic field strength of 120 mT, the robot demonstrates segmented peristaltic motion. Specifically, the magnetic field is initially applied to the distal end of the structure, inducing localized compressive deformation and increasing the internal pressure of the viscous fluid (yogurt). As the driving magnetic field progresses along the robot, the deformation region propagates accordingly, forming a traveling peristaltic wave that propels the fluid toward the proximal end, effectively replicating the peristaltic transport observed in the natural intestinal system (Supplementary Fig.24b and Movie 10).

The magnetic moment distribution of the wave-like peristaltic robot is shown in Supplementary Fig.24a, where the overall magnetic moment density rotates by 9° between adjacent slices within the x–y plane. Owing to the specific magnetization profile assigned to each layer, the slices can periodically contract and expand with a defined phase shift under a rotating magnetic field applied in the x–y plane. This coordinated deformation generates a traveling wave along the tubular soft pump, enabling wave-like peristaltic motion (Supplementary Fig.24c and Movie 11).

**Supplementary Note 5. Simulation of 3D large-scale uniform magnetic field generator**

The simulation was conducted using COMSOL Multiphysics, specifically employing the following modules: 1. Magnetic Fields (mf): This module was used to simulate the spatial distribution of the static magnetic field generated by permanent magnets and to analyze the flux density vector field in the printing region. 2. Infinite Element Domain: Implemented to define the outer boundary of the computational domain and to ensure accurate decay of the magnetic field at far distances.

Geometry and Domain Definition: Two rectangular permanent magnets were modeled as 40 mm × 40 mm × 20 mm cuboids, symmetrically placed on either side of the cylindrical printing region, with the center at coordinates (0, 0, 0). The magnets were placed along the horizontal axis and assigned a variable rotation angle of –α, initialized as 0°. The entire simulation domain was embedded within a 160 mm × 80 mm × 80 mm bounding box. All six sides were assigned 10 mm-thick infinite element layers to approximate an open boundary condition (Supplementary Fig. 25).

Material Properties. Permanent magnets (N35 sintered NdFeB): Assigned to the two magnet domains, with remanent flux density *B_r_* = 1.2 T, relative permeability *μ_r_*= 1.05. Air: Assigned to all other domains (including the printing area and surroundings) using the predefined “Air” material from the COMSOL material library, with *μ_r_* = 1

Physics Settings: The Magnetic Fields (mf) interface with No Currents was selected. A Magnetic Flux Conservation feature was applied specifically to the permanent magnet domains to simulate remanent magnetization effects.

Boundary Conditions: The outer surfaces of the infinite element layer were treated as magnetically insulating boundaries. The continuity of magnetic flux across internal

Validation Metrics: The simulation results (magnetic flux density distribution) were validated by comparing them to experimental measurements using Hall effect sensors in the printing region. Agreement was evaluated in terms of the angular orientation of magnetic field lines and the field strength distribution, ensuring deviations of less than ±10% from experimental measurements.

**Supplementary Note 6. Simulation of magnetic actuated deformation**

Finite Element Analysis (FEA) of the robot’s deformation under magnetic actuation was conducted using ABAQUS/Standard. A user-defined element (UEL) subroutine was employed to model the magnetic-mechanical coupling behavior of magnetically responsive soft materials. This subroutine was originally proposed by Zhao[3] et al. and modified in this study to suit the simulation of soft magnetic structures under uniform magnetic fields. This UEL routine, calculates the magnetic torque based on the cross product between the remanent magnetization vector *Mr* and the external magnetic field H, following the relation:

$$\begin{aligned} \tau=\mu_{0}(M_{r}\times H)\#\left( S7 \right) \end{aligned}$$

Each simulation element was assigned a fixed magnetization direction, and the subroutine transformed the computed torques into equivalent nodal forces that drive mechanical deformation. These forces were then passed into ABAQUS for equilibrium calculation under nonlinear large deformation settings. The subroutine supports time-varying magnetic fields and iteratively updates the deformation until quasi-static equilibrium is reached.

Material Properties: Young’s modulus: 𝐸 = 1 MPa, representing the elasticity of the soft matrix. The material density was set to 4.2 g/cm³, and the Poisson’s ratio was assigned a value of 0.49.

Magnetic flux density: An actuation magnetic field of 150 mT was applied uniformly. For magnetic robots, a magnetic flux density of 9.5 KA/m was used to represent the embedded remanent magnetization.

Boundary Conditions: The base of the printed structure was fixed to replicate experimental clamping conditions. The rest of the structure was free to deform under the influence of the applied magnetic torque computed in the UEL routine.

Validation Metrics: The simulated deformation profiles and displacements were compared against experimental observations under identical magnetic actuation conditions. Good agreement was observed in both displacement amplitude and shape evolution, confirming the accuracy of the model (Supplementary Fig. 26).

**Supplementary Figures**


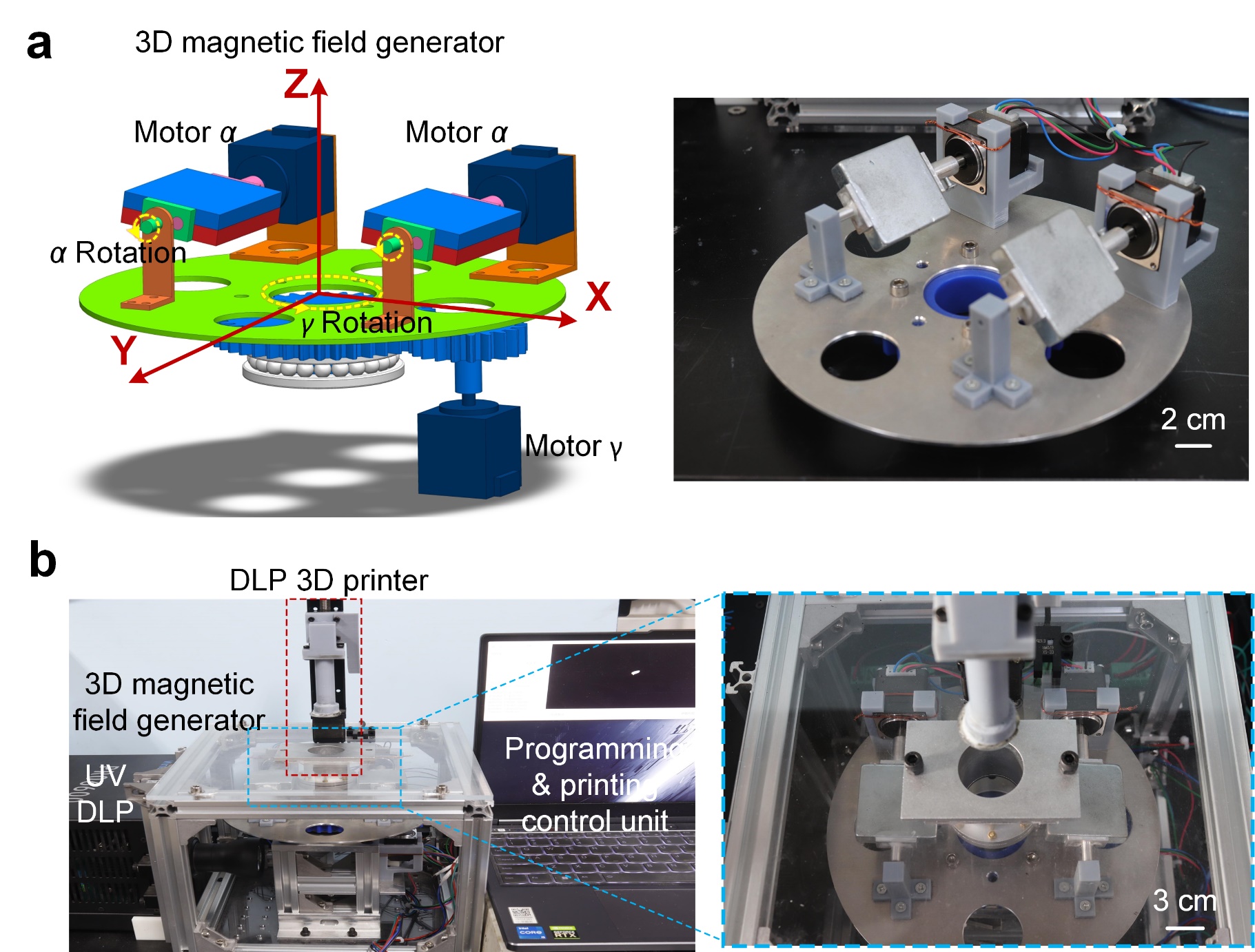


**Supplementary Fig. 1 Structure of *in situ* pixel-scale magnetic programming 3D printing device (Supplementary Movie 1).** a) The 3D magnetic field generator. b) The integration of magnetic field generator and DLP 3D printer.


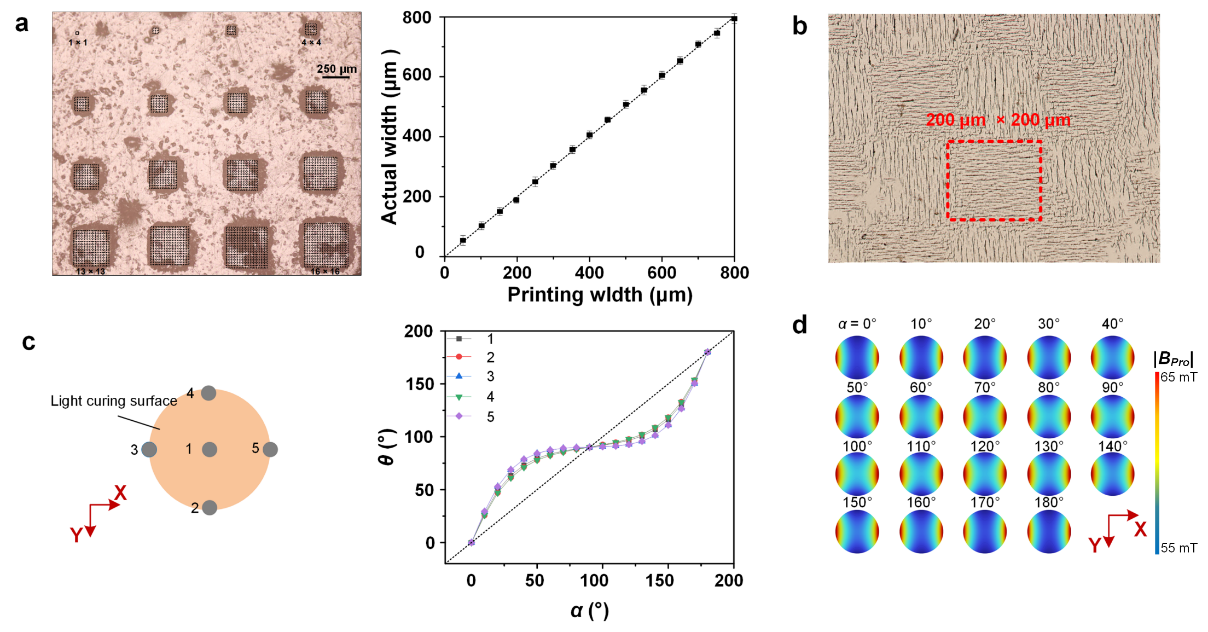


**Supplementary Fig. 2 Basic equipment parameters**. a) UV curing printing accuracy. b) Magnetic programming resolution of the printing equipment. c) Printing angle uniformity at different points within the print area. d) Simulation of magnetic field strength uniformity at different *α.*


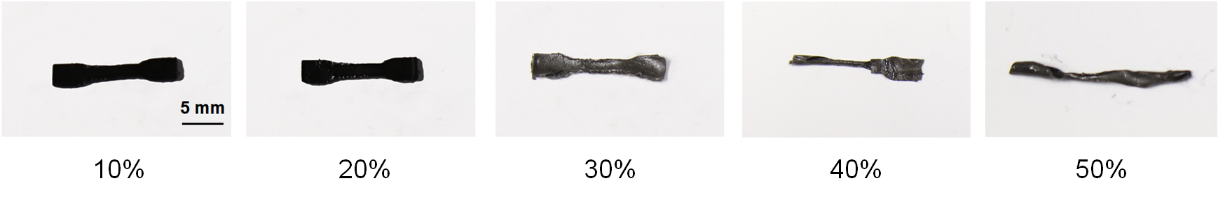


**Supplementary Fig. 3 Determination of mass fraction of magnetic particles.** When the mass fraction exceeds 20%, the curing UV light transmittance is greatly reduced and leads to incomplete printed structure.


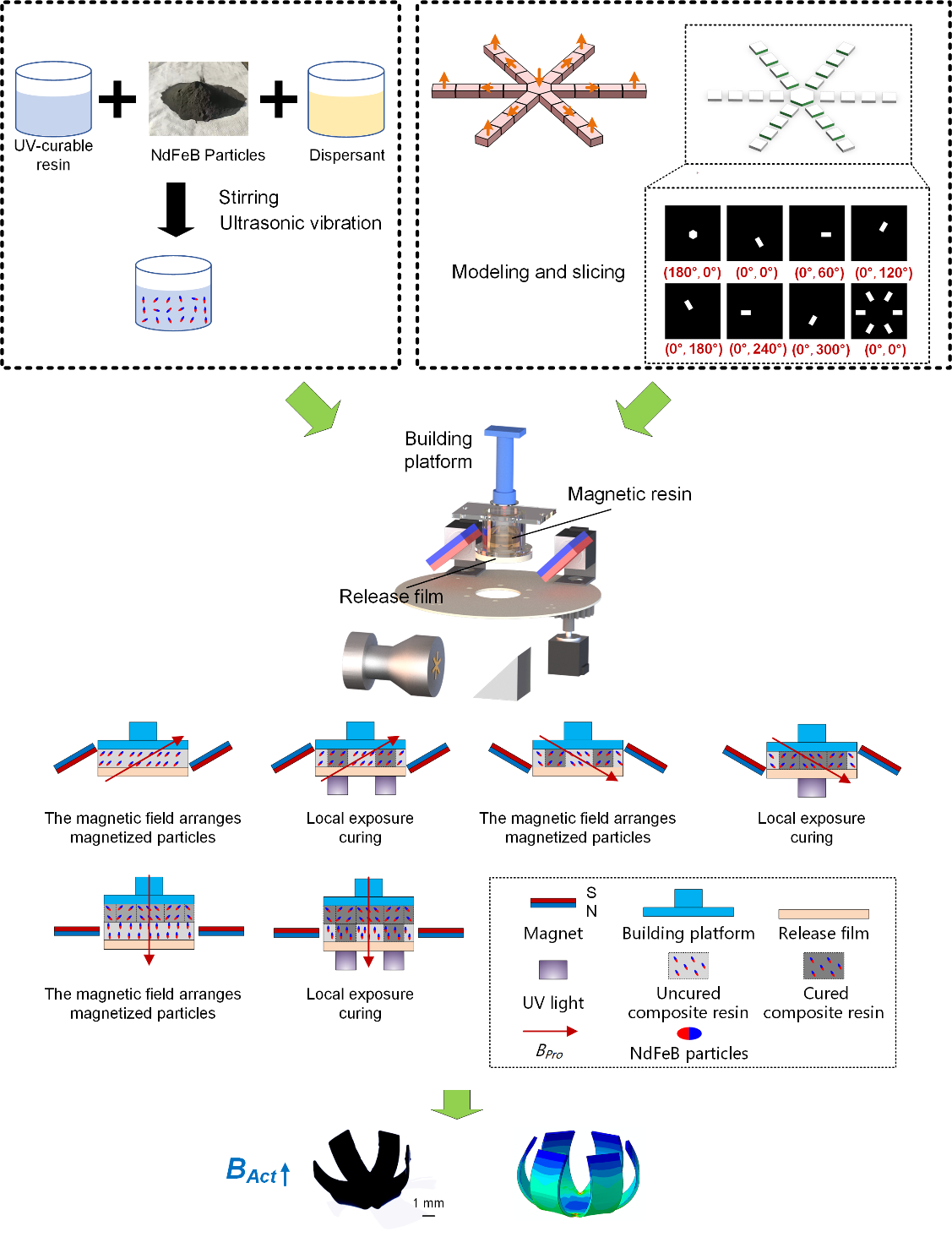


**Supplementary Fig. 4 Complete printing process.** a) Processing of printed materials. b) Zoning of the printed model according to the direction of magnetization. c) Printing process. d) Printed robot.


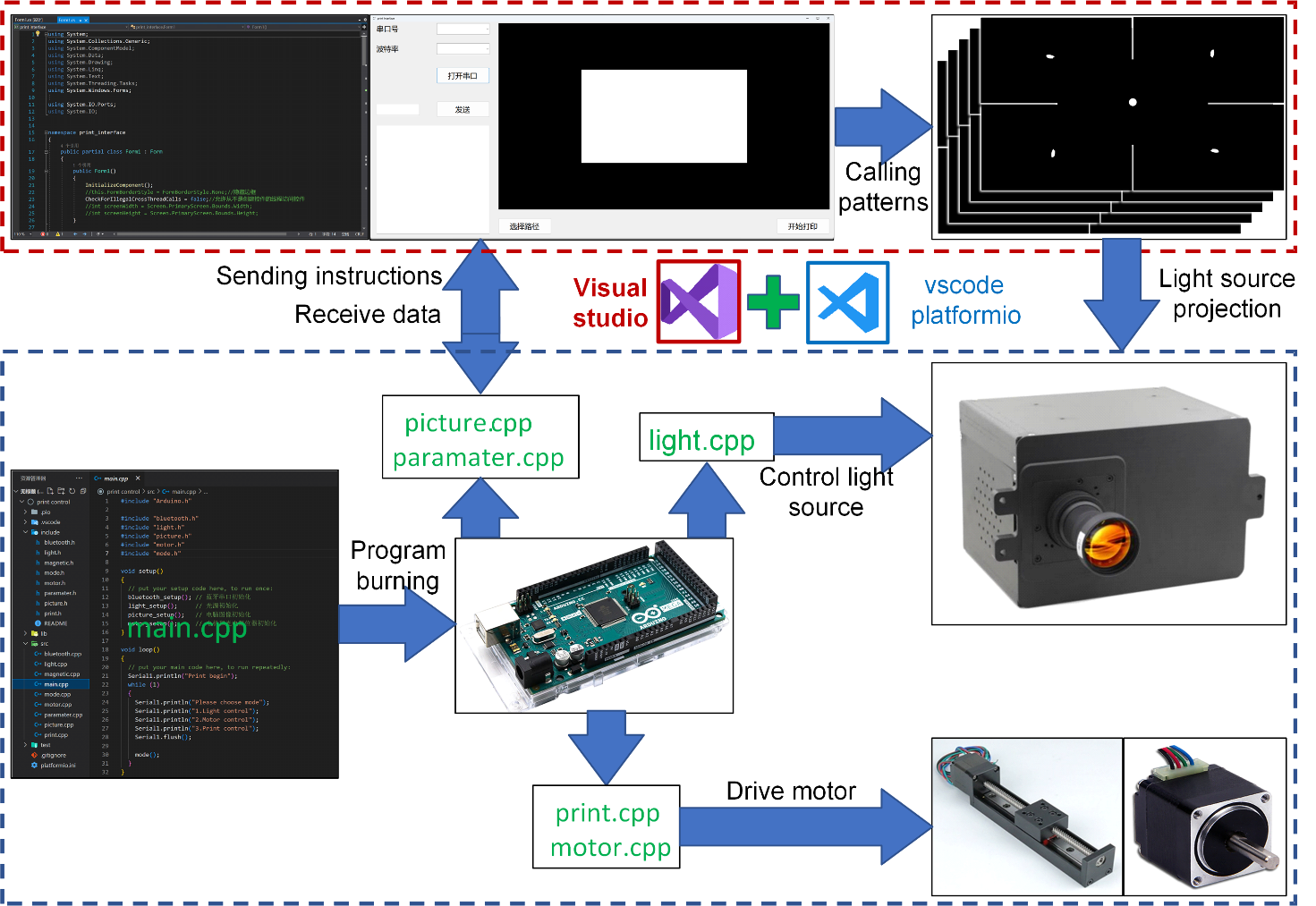


**Supplementary Fig. 5 Software and hardware work process of the printing device.**


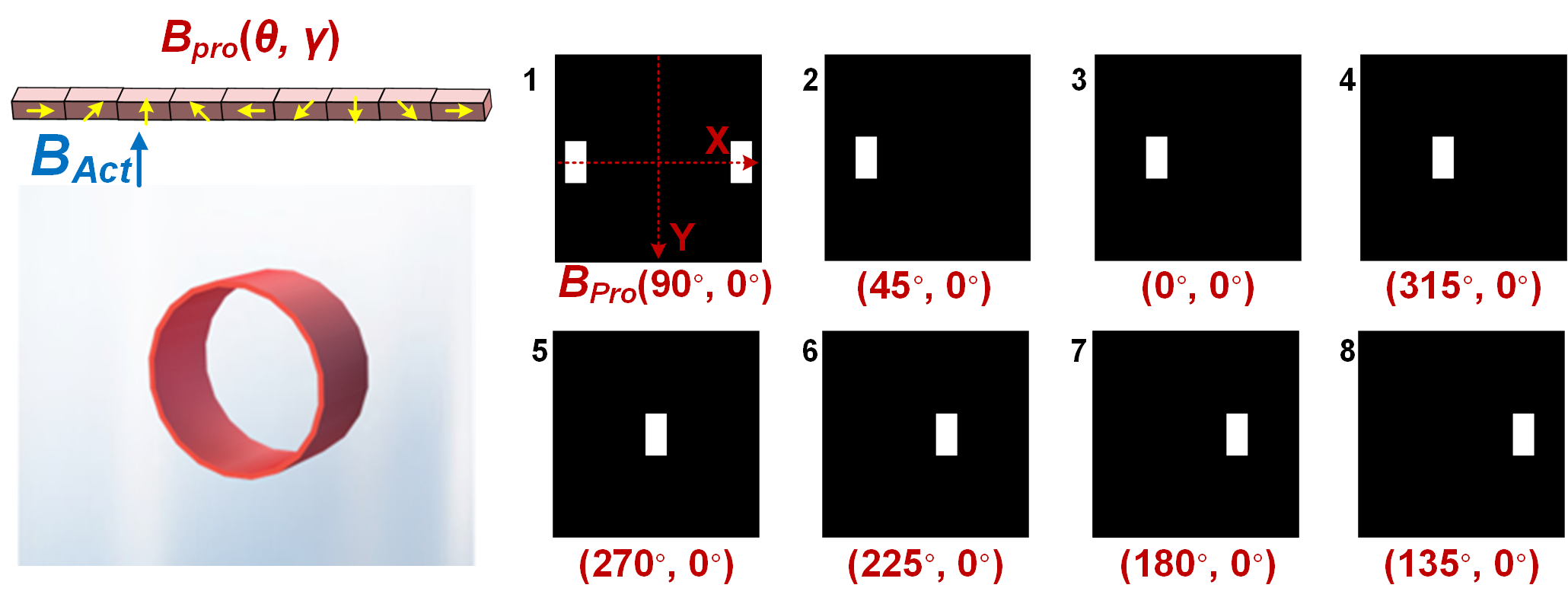


**Supplementary Fig. 6 UV light curing and magnetic field coupling processes in millirobot printing.** Slicing patterns of the rolling robot.


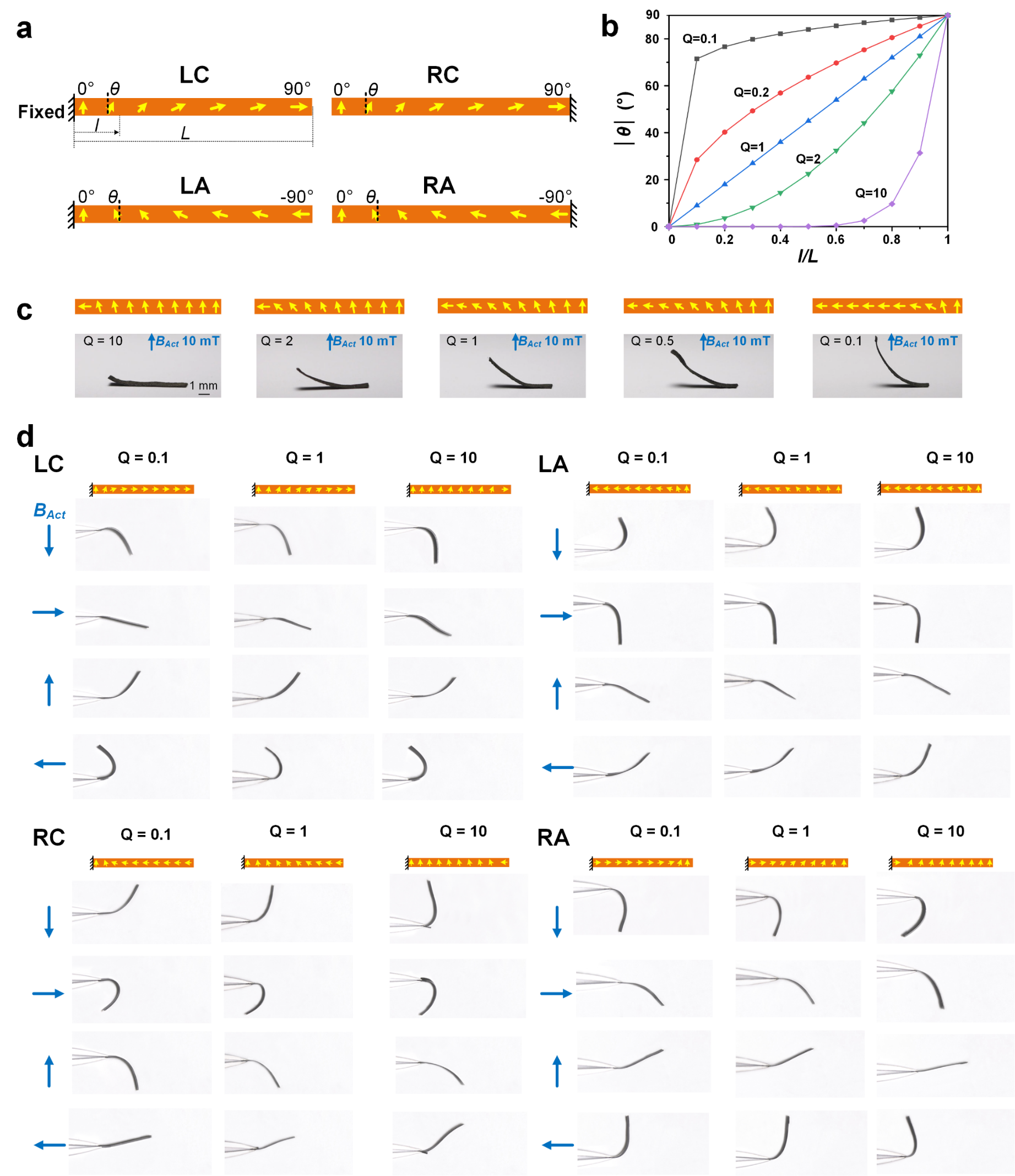


**Supplementary Fig. 7 The cantilever strip’s magnetization design and deformation**. a) Four basic forms of deformation for cantilever strip. With left end fixed, *θ* rotates clockwise from 0 degrees to 90 degrees (LC). With right end fixed, *θ* rotates clockwise from 0 degrees to 90 degrees (RC). With left end fixed, *θ* rotates anticlockwise from 0 degrees to 90 degrees (LA). With right end fixed, *θ* rotates anticlockwise from 0 degrees to 90 degrees. b) *Q* represents the changing rate of *θ* in cantilever strip. A more accurate control of bending deformation can be realized by adjusting *Q*. c) Applying a vertically upward magnetic field, different Q corresponds to different degrees of deformation. d) LC, LA, RC and RA with *Q* = 0.1, 1 and 10 exhibit different deformation under 4 direction actuating magnetic field, which provides sufficient example for key-nodes splicing magnetizing method.


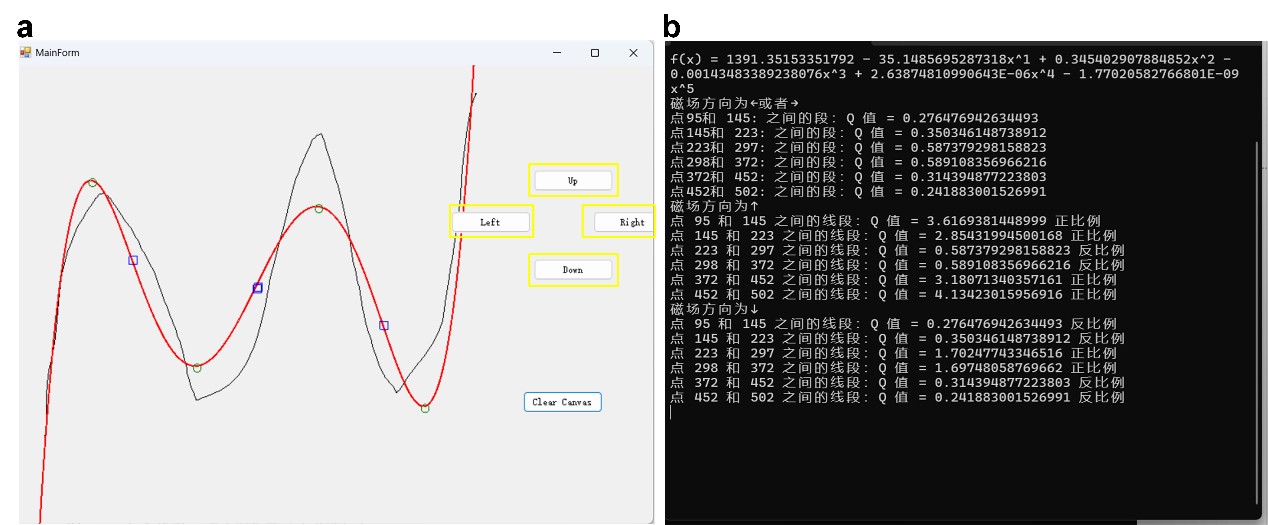


**Supplementary Fig. 8 1D arbitrary shape division software.** a) Software screenshots. b) Each segment after division.


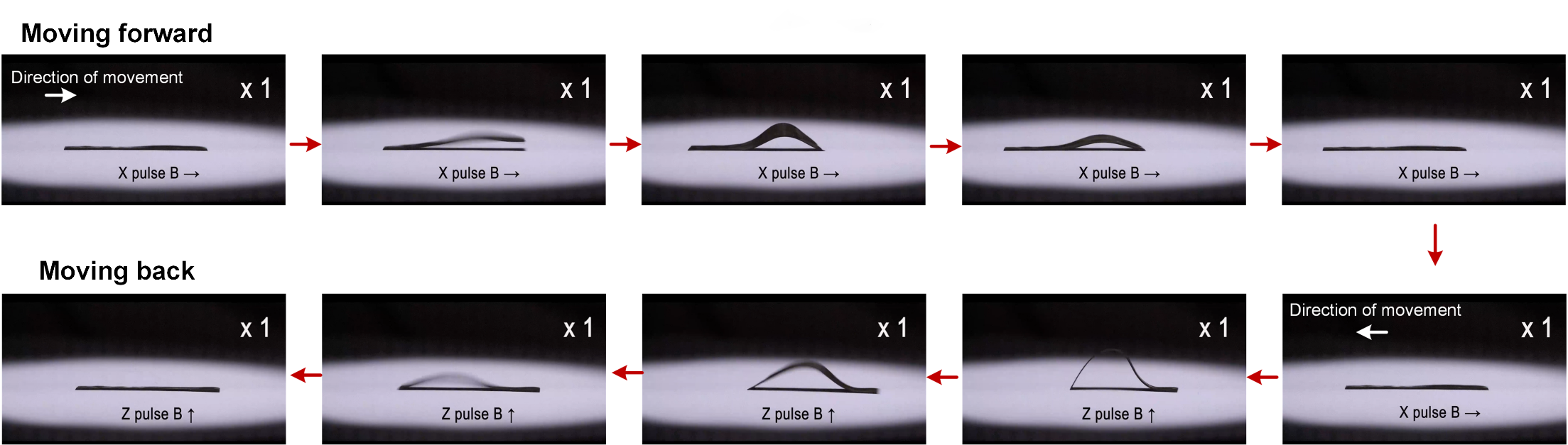


**Supplementary Fig. 9 Caterpillar-like robot demonstrating bidirectional motion (Supplementary Movie 4).** Under the action of pulsed magnetic fields, the caterpillar-like robot exhibits two-way locomotion: it crawls forward when *B_Act_* is applied along the X direction, and crawls backward when *B_Act_* is applied along the Z direction


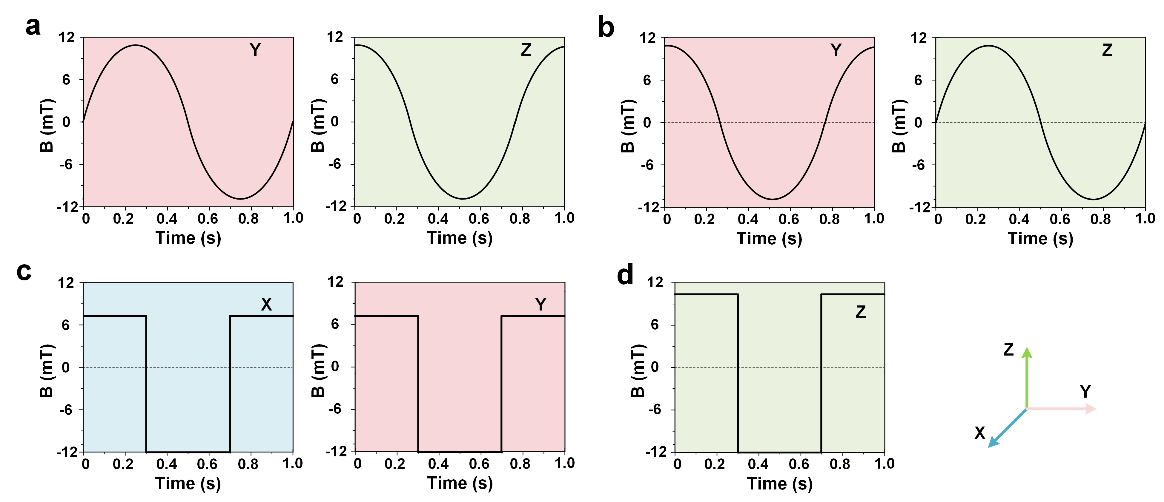


**Supplementary Fig. 10 Actuation magnetic fields of different motion modes in Figure 4.** a) For the peristaltic state, two sinusoidal magnetic fields along the Y-axis and Z-axis combine to form a rotating magnetic field in the YZ plane, with the initial magnetic field direction aligned with the positive Z-axis. b) For the rolling state, two sinusoidal magnetic fields along the Y-axis and Z-axis combine to form a rotating magnetic field in the YZ plane, with the initial magnetic field direction aligned with the positive Y-axis. c) For the forward crawling state, pulsed magnetic fields of equal strength are applied along the X-axis and Y-axis to form a composite magnetic field at a 45°angle to the positive direction of the X-axis. d) For the backward crawling state, a pulsed magnetic field is applied along the Z-axis.


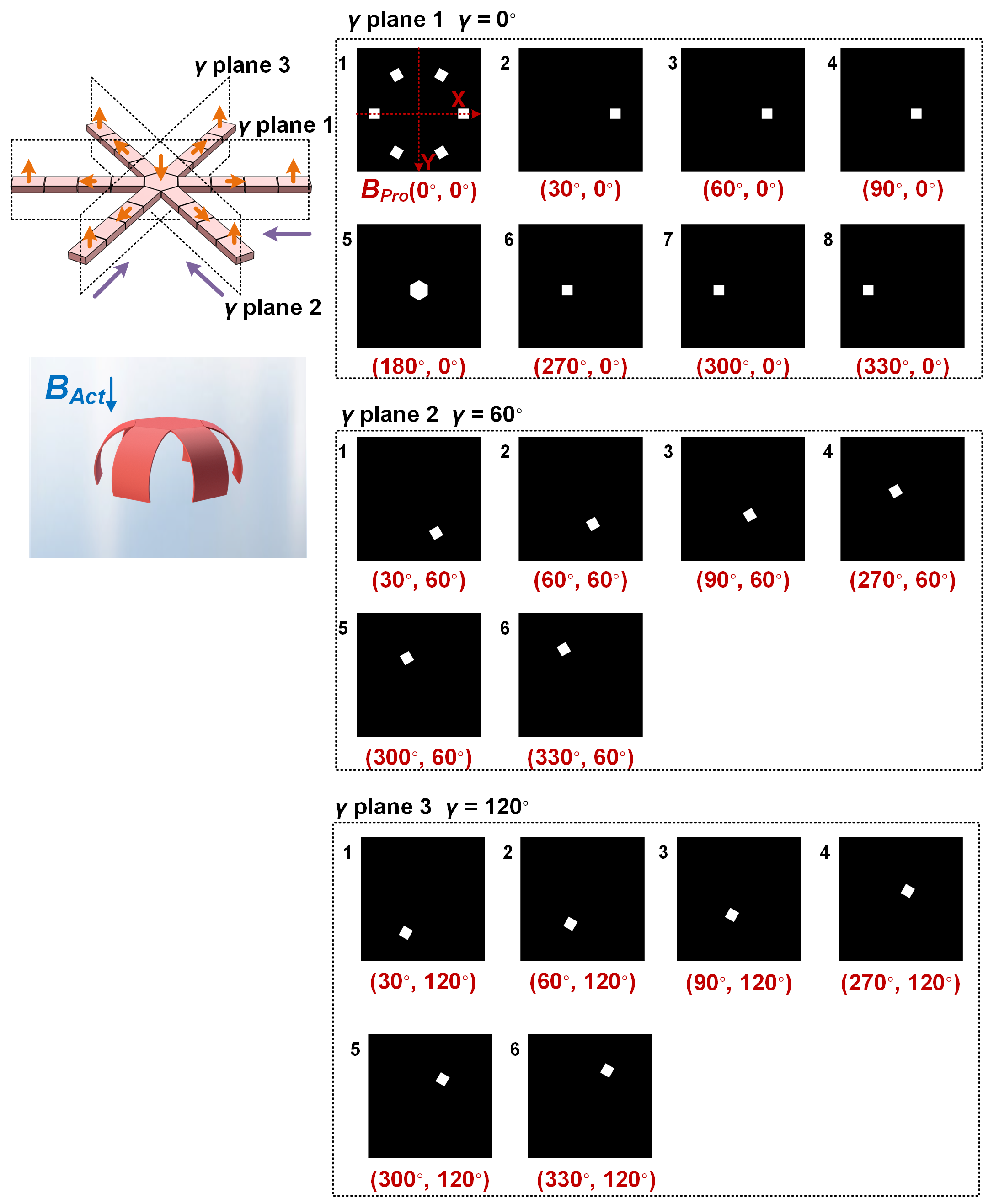


**Supplementary Fig. 11 UV light curing and magnetic field coupling processes in millirobot printing.** Slicing patterns of the six-arm robot.


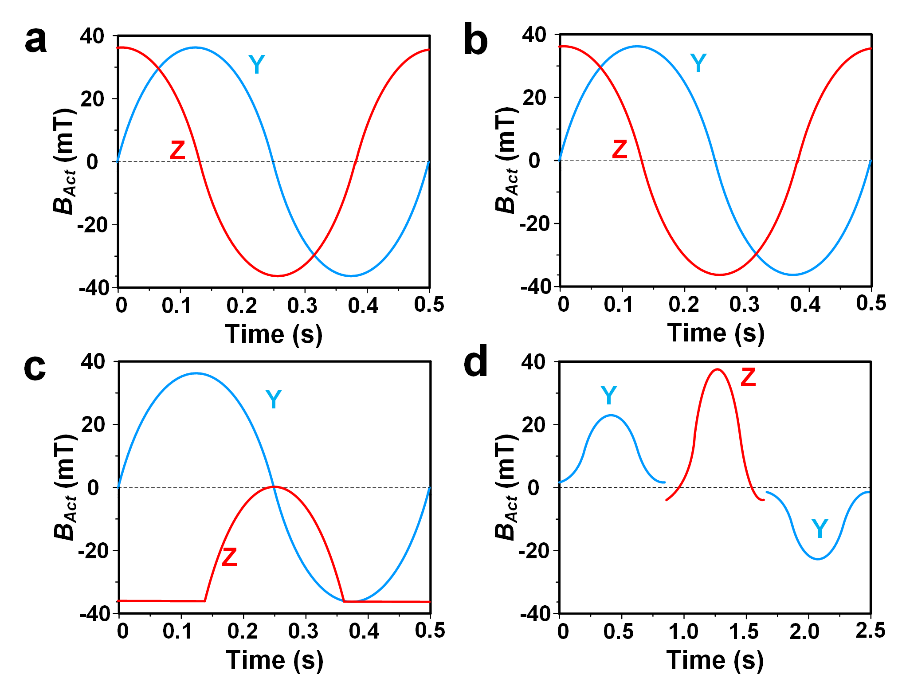


**Supplementary Fig. 12 Actuation magnetic fields of different motion modes in Figure 4.** a) For the rolling state, the two sinusoidal magnetic fields of Y axis and Z axis are combined to form the rotating magnetic field on the YZ plane. b) For obstacle crossing state, apply the rotating magnetic field of YZ plane. c) For crawling state, Y-axis and Z-axis magnetic fields are compounded. d) For the swimming state, the corresponding magnetic field is applied in the Y-axis direction, and the small phase transition moves in the horizontal direction. Apply the corresponding magnetic field in the Z-axis direction, and the large phase transition moves in the vertical direction.


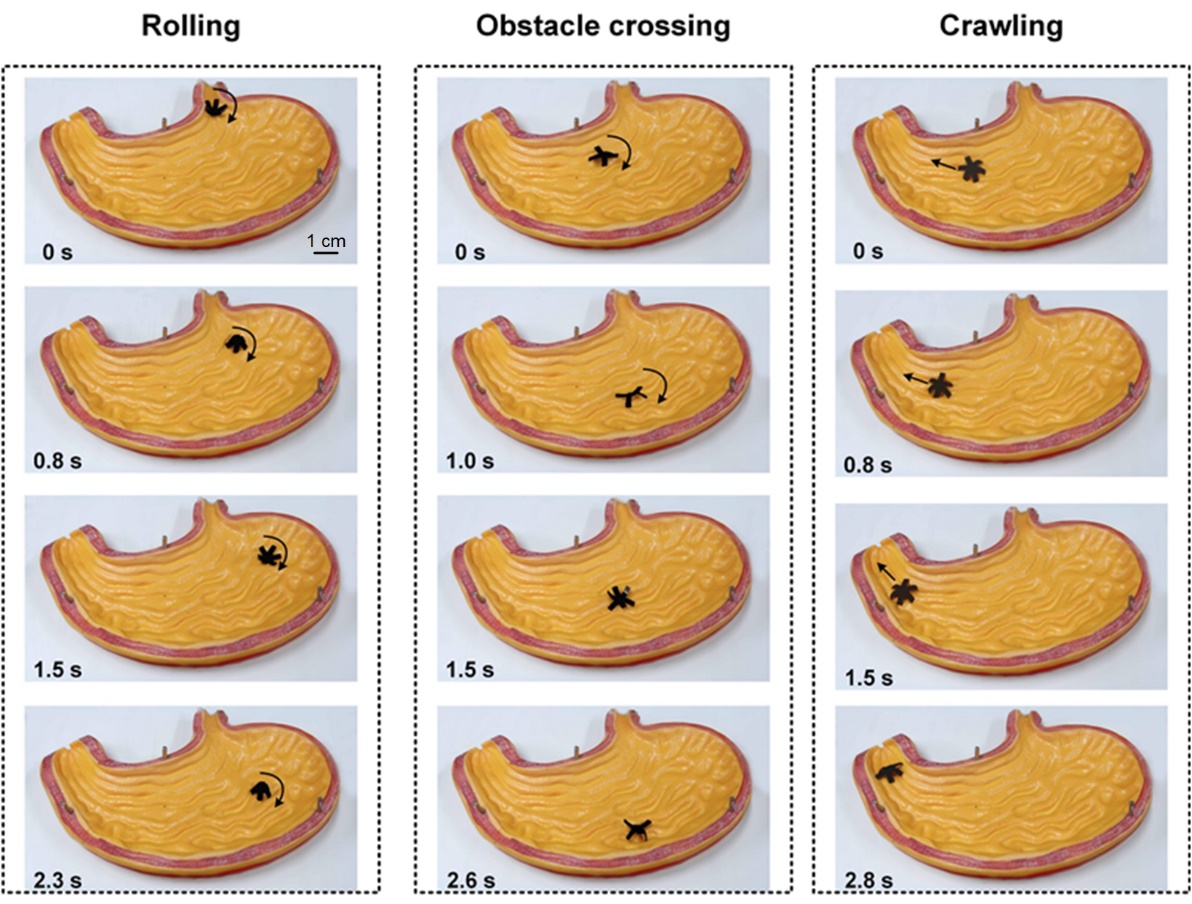


**Supplementary Fig. 13 Different six claw robots moving in the stomach (Supplementary Movie 5).**


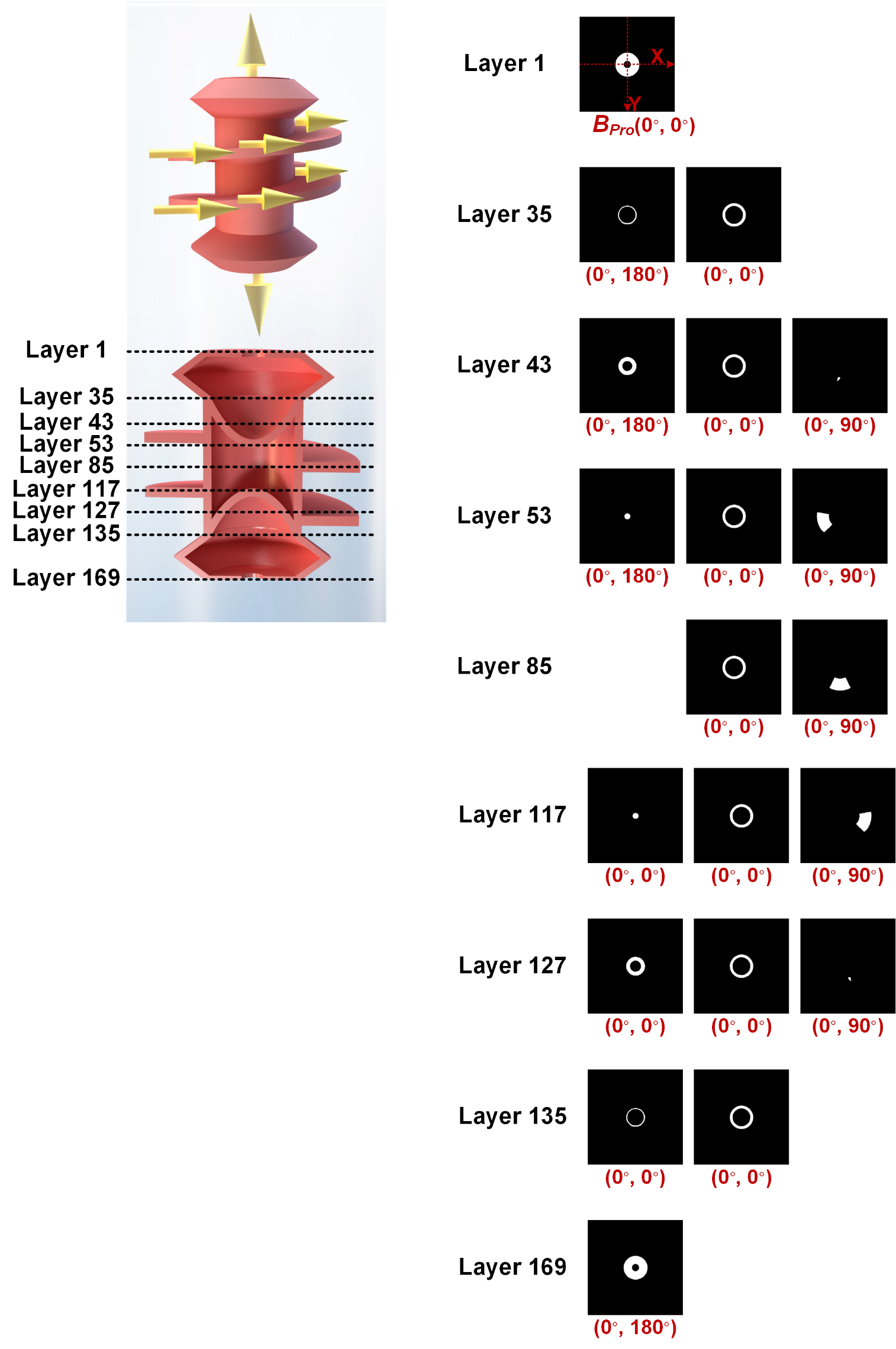


**Supplementary Fig. 14 UV light curing and magnetic field coupling processes in millirobot printing.** Slicing patterns of the spiral capsule robot.


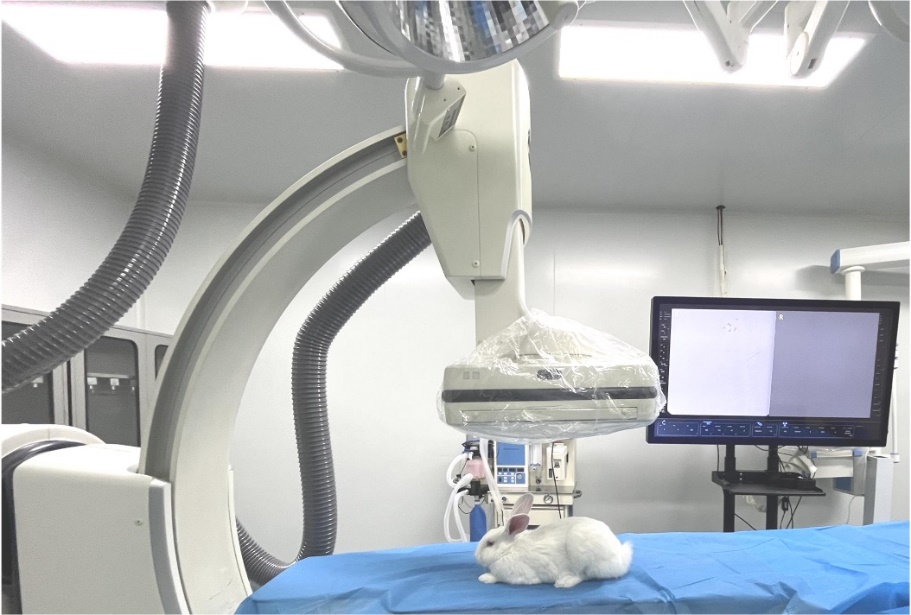

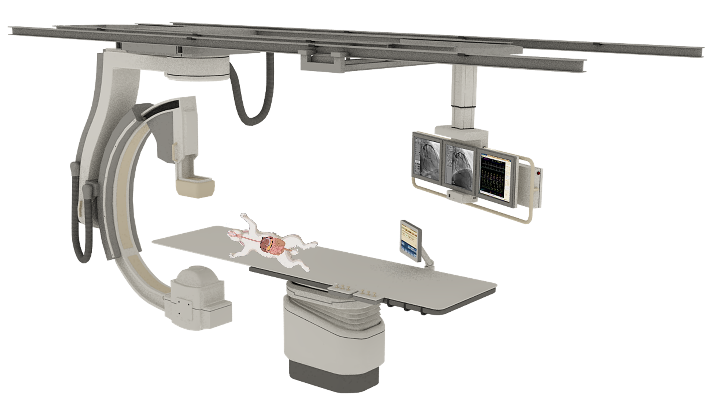


**Supplementary Fig. 15 The experimental setup for the magnetic actuation of the modularized microrobot that is guided by the X-ray image.**


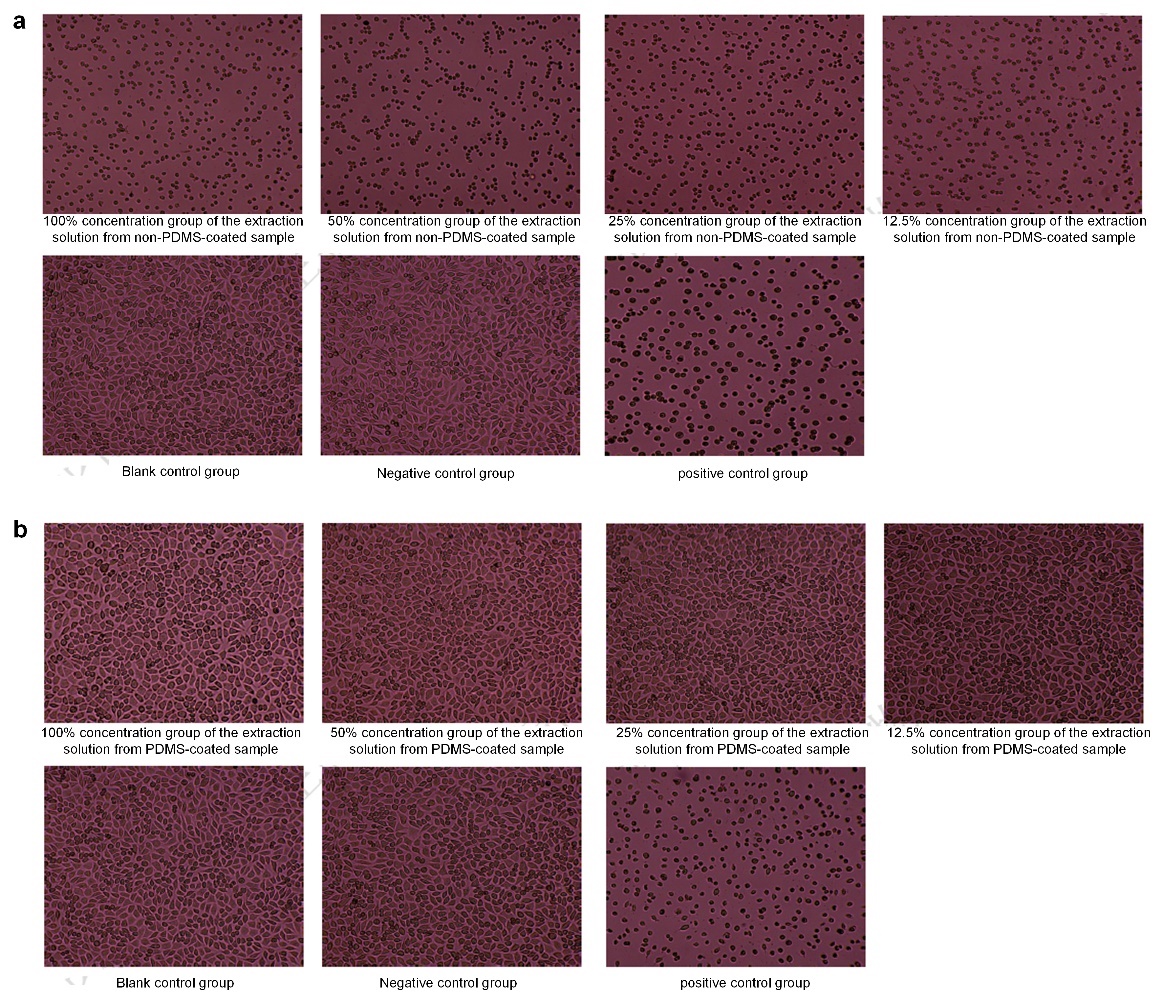


**Supplementary Fig. 16** **In vitro cytotoxicity evaluation of magnetic robots using MTT assay.** a) Non-PDMS-coated magnetic robots: L929 fibroblasts exhibited severe morphological alterations (cytotoxicity grade 4), with significantly reduced viability (14.2%, below the 70% cytotoxicity threshold), indicating high toxicity. b) PDMS-coated magnetic robots: cells maintained normal morphology (cytotoxicity grade 0), and viability remained high (79.3%, above the 70% threshold), demonstrating effective biocompatibility.


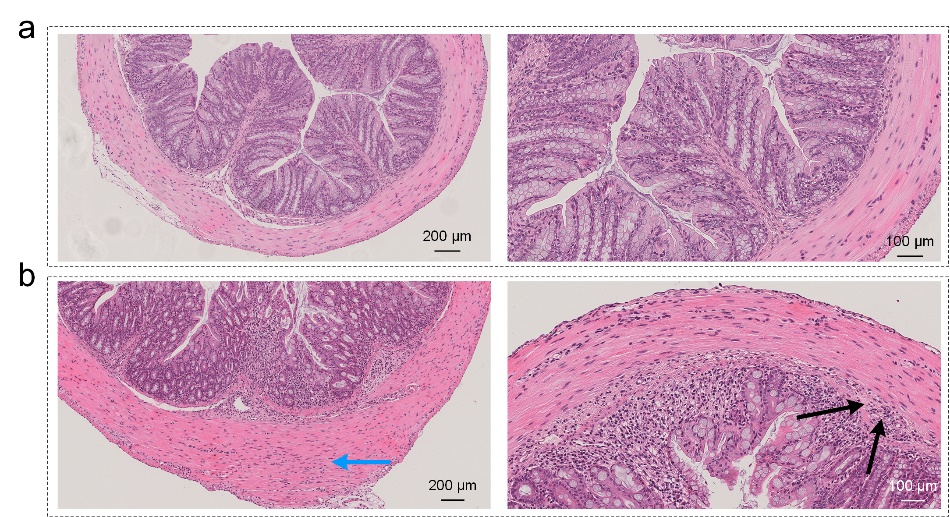


**Supplementary Fig. 17 Histological analysis of rat intestine after oral administration of magnetic robots.**  a) PDMS-coated: Intact mucosa and crypts, no necrosis; inflammation score: 1.3 ± 0.4. b). Non-coated: Wall thickening (blue), submucosal immune infiltration (black), signs of inflammation; inflammation score: 4.2 ± 0.8.


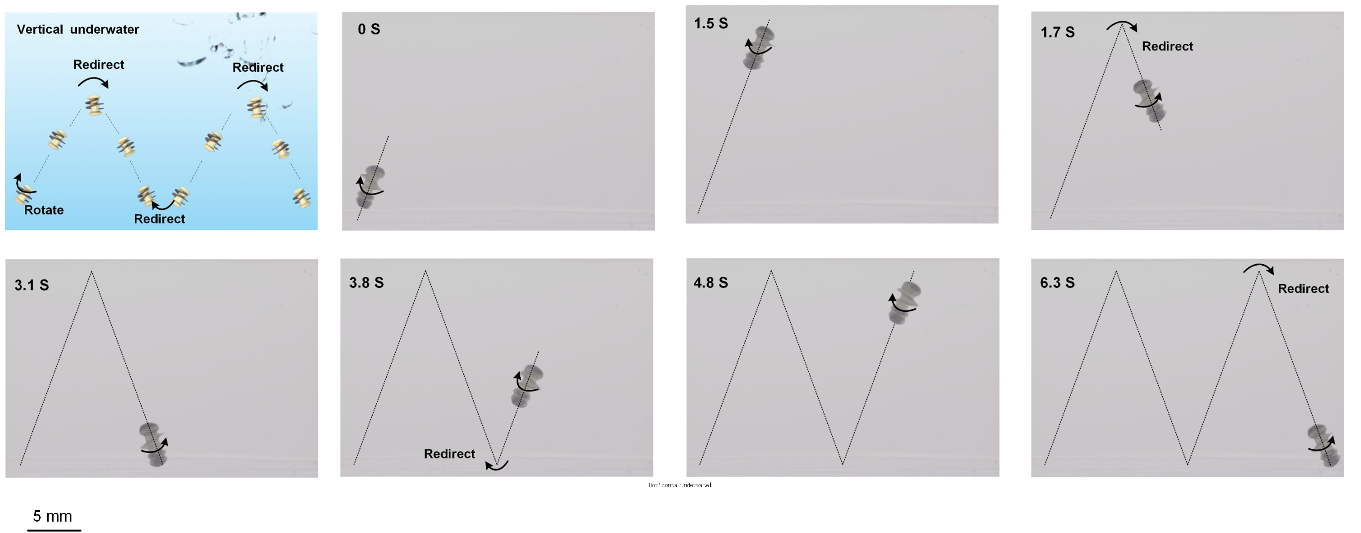


**Supplementary Fig. 18 M-shaped motion of robots in the vertical direction (Supplementary Movie 9).**


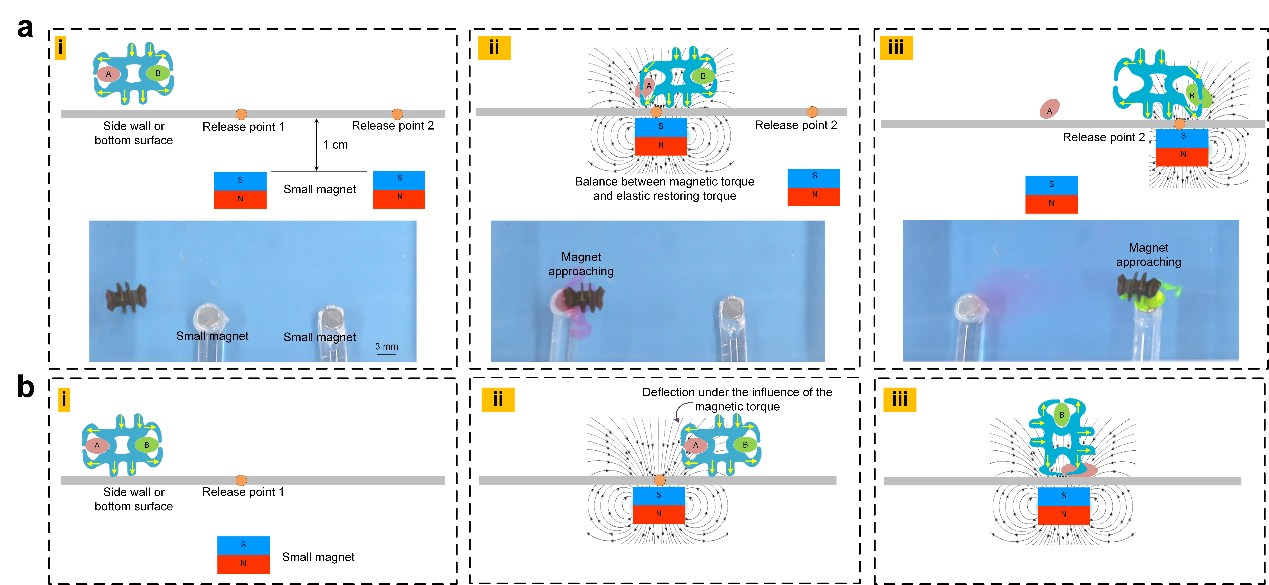


**Supplementary Fig. 19 Deformation of the magnetic robot during cargo release.** a) The deformation occurs when the magnet approaches the robot at a position between the compartment and the helical structure. The compartment reaches a force equilibrium under the magnetic field, compressing and releasing the drug. b) The robot flips when the magnet acts directly on the thin-walled compartment, potentially affecting subsequent movement.


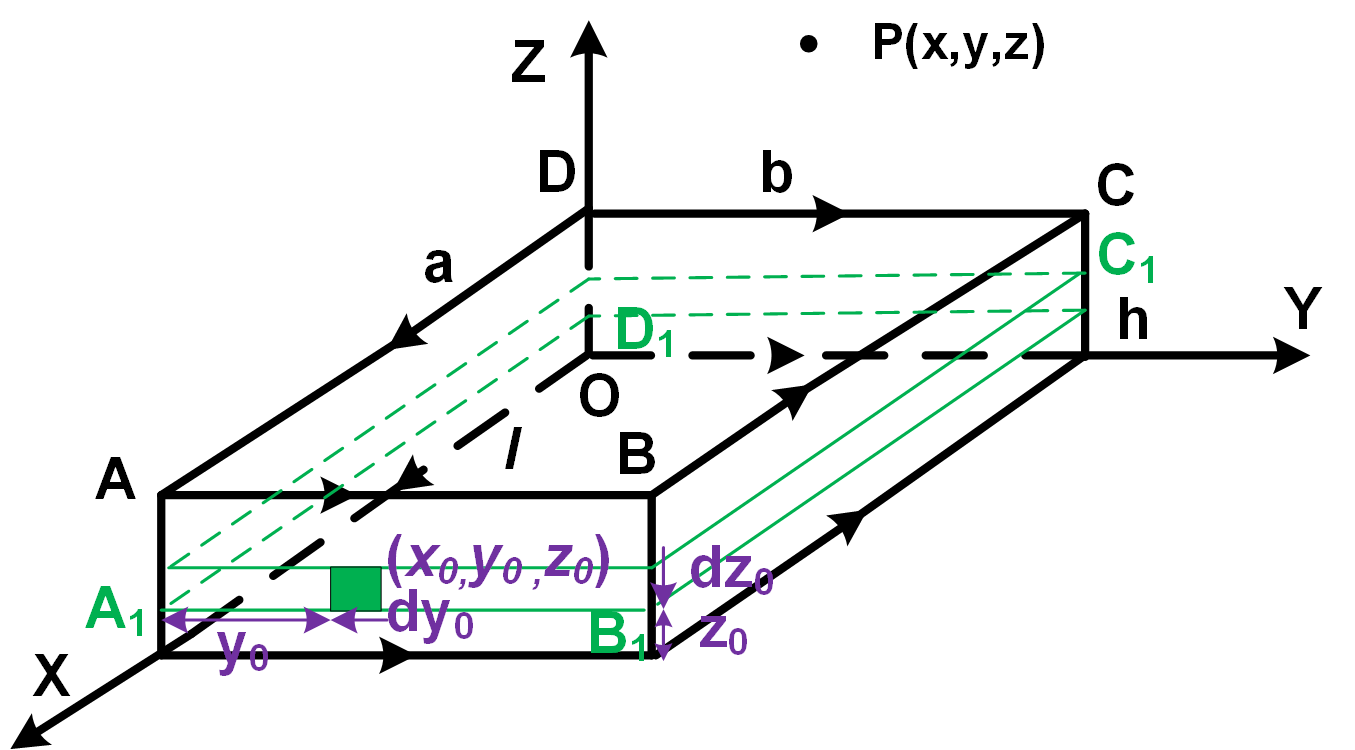


**Supplementary Fig. 20 Rectangular magnet model using molecular current.**


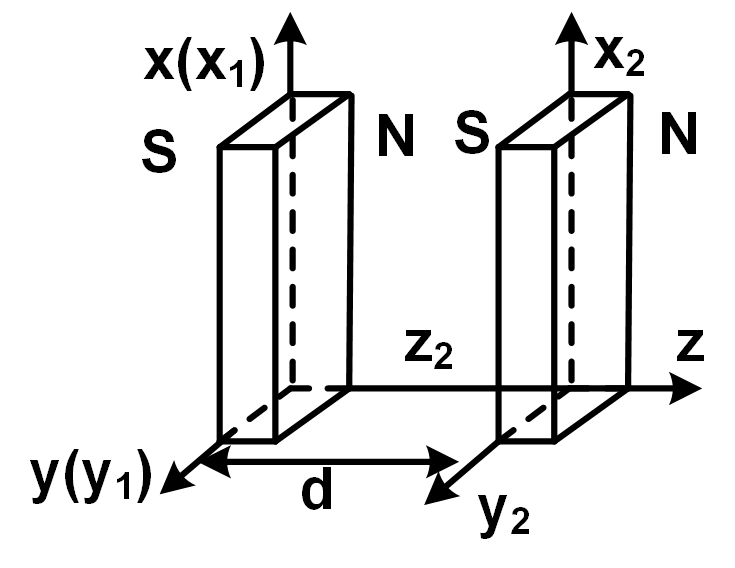


**Supplementary Fig. 21 Double magnets in the same direction.**


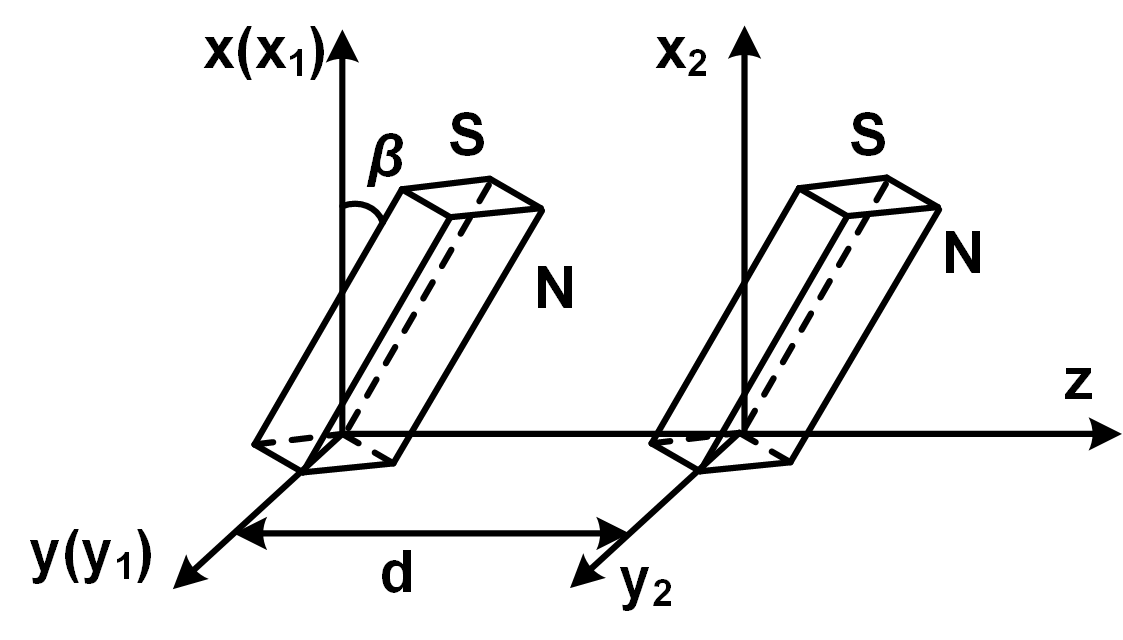


**Supplementary Fig. 22 Double magnets with the same *α.***


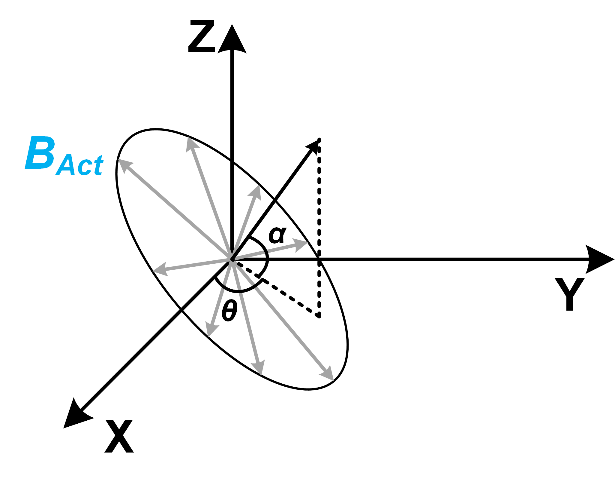


**Supplementary Fig. 23 3D actuating magnetic field generated by three-axis Helmholtz coils.**


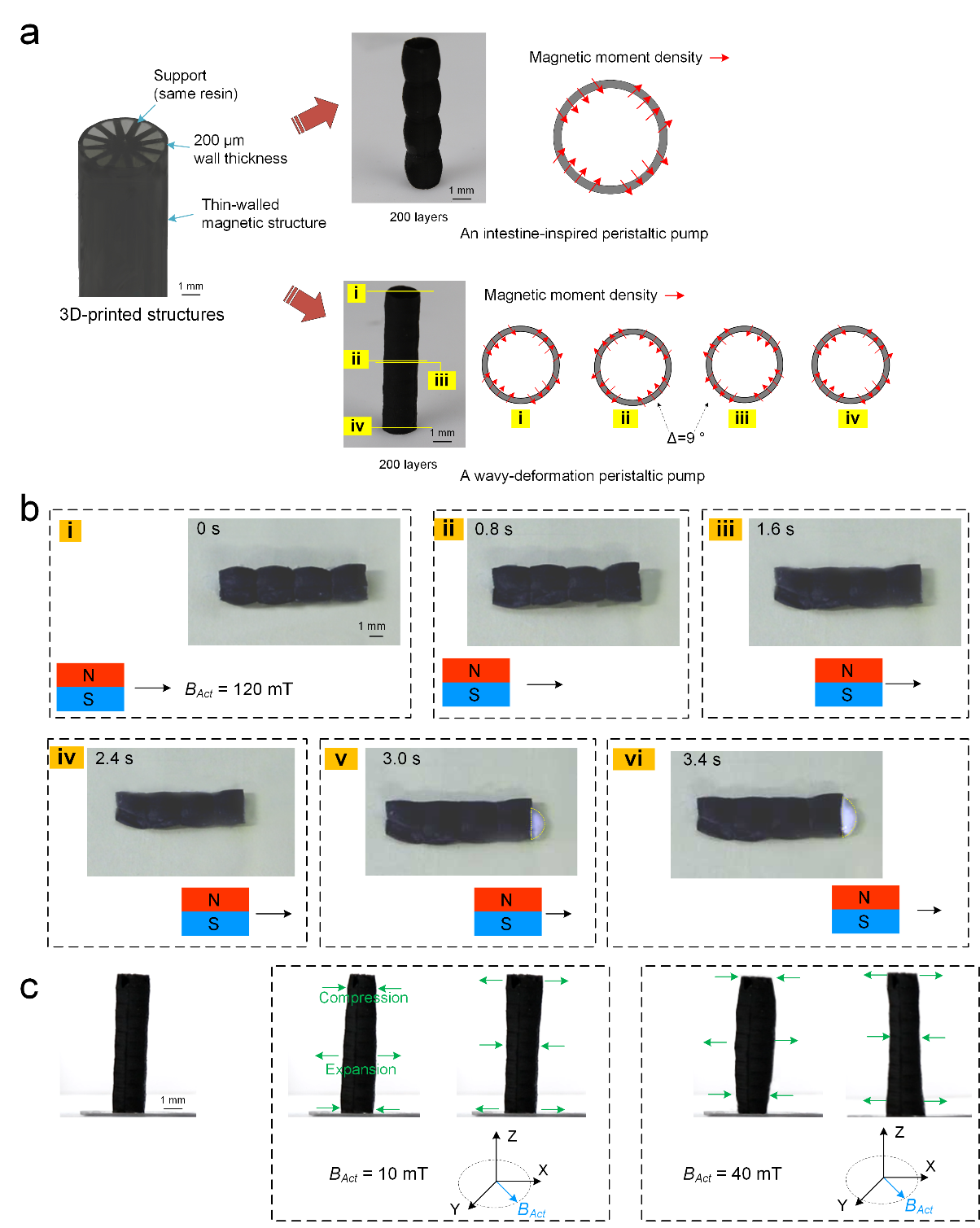


**Supplementary Fig.24** **3D-printed thin-walled structures (Supplementary Movie 10, 11).** a) Two types of printed thin-walled tubular structures. b) Intestine-inspired peristaltic motion. c) Wave-like peristaltic pump motion.


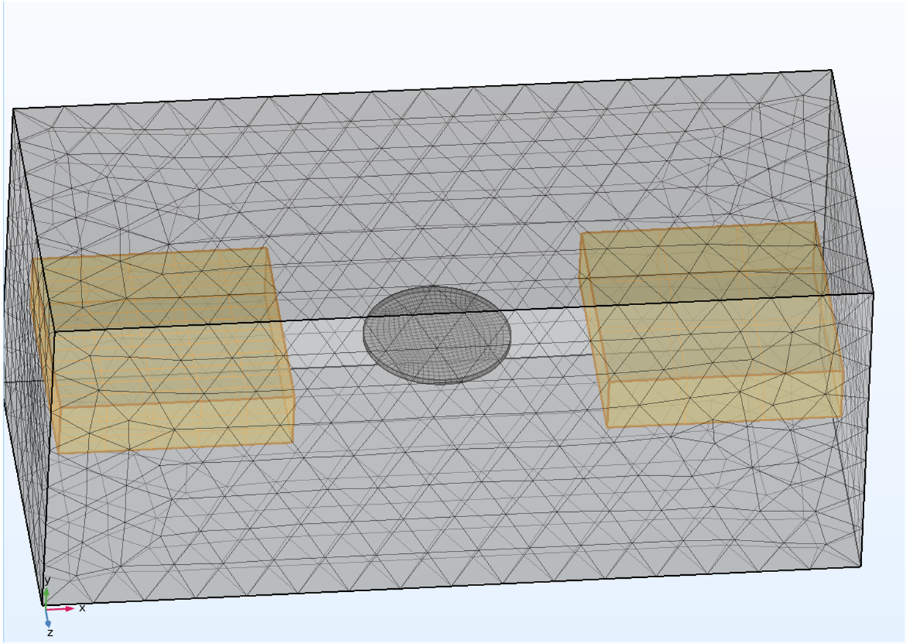


**Supplementary Fig. 25 COMSOL simulation design of magnetic field generator: geometric model design and mesh generation.**


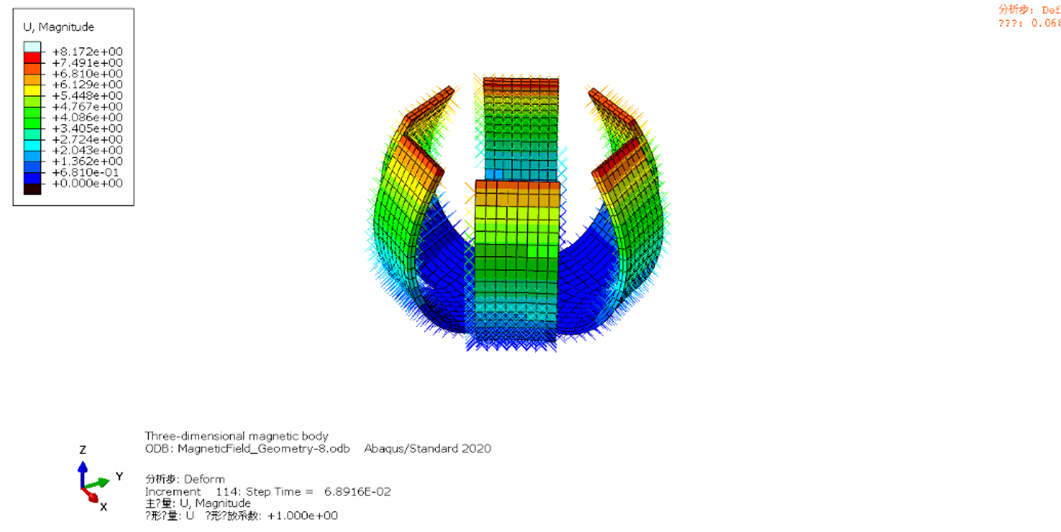


**Supplementary Fig. 26 The design of deformation simulation for magnetic robots using Abaqus: geometric model design and mesh generation.**

**Table S1.** Comparison of additive manufacturing methods and applications for magnetic soft robots.

| Magnetic particles | Magnetizing methods | Magnetic field property | Methods | Structure resolution | Magnetization arrangement | Reference |
| --- | --- | --- | --- | --- | --- | --- |
| **NdFeB particles** | **Two fixed parallel permanent magnet** | **Uniform 3D**  **60 mT** | **Digital light processing (DLP)** | **50 μm** | **3D** | **This work** |
| Flakes coated by Fe_3_O_4_ nanoparticles | One axis single permanent magnet | Gradient 2D  40 mT | Direct ink-writing (DIW) | 0.8 mm | 2D | [4] |
| Flakes coated by Fe_3_O_4_ nanoparticles | One axis single permanent magnet | Gradient 2D  15 mT | Drop-on-demand (DOD) | 0.33 mm | 2D | [5] |
| Flakes coated by Fe_3_O_4_ nanoparticles | Three-axis Helmholtz coils | Uniform 3D | Photocuring | 90 μm | 3D | [6] |
| FeNi nanoparticles | Two fixed parallel permanent magnet | Uniform 1D | Laser photocuring | / | 1D | [7] |
| Fe_3_O_4_ nanoparticles | Two fixed parallel permanent magnet | Uniform 1D 10 mT | Photocuring | 100 μm | 1D | [8] |
| NdFeB particles | One oriented magnetic field applied to the nozzle | Gradient 1D  50 mT | Direct ink-writing (DIW) | 80 μm | 2D | [3] |
| NdFeB particles | Single permanent magnet | Gradient 3D  20 mT | Digital light processing (DLP) | 100 μm | 3D | [9] |
| CrO_2_ | Single permanent magnet | Gradient 3D  15 mT | Laser cutting | 38μm | 3D | [10] |

**Table S2.** Cell cytotoxicity test about non-PDMS-coated magnetic robot.

|  | **100% concentration group** | **50% concentration group** | **25% concentration group** | **12.5% concentration group** | **Negative control group** | **Positive control group** |
| --- | --- | --- | --- | --- | --- | --- |
| **Mean survival rate (%)** | 14.2% | 14.6% | 15.1% | 15.9% | 99.7% | 14.2% |

**Table S3.** Cell cytotoxicity test about PDMS-coated magnetic robot.

|  | **100% concentration group** | **50% concentration group** | **25% concentration group** | **12.5% concentration group** | **Negative control group** | **Positive control group** |
| --- | --- | --- | --- | --- | --- | --- |
| **Mean survival rate (%)** | 79.7% | 86.3% | 87.7% | 92.5% | 99.6% | 16.1% |

**Supplementary Videos**

Supplementary Video 1.

The printing process of the *in situ* pixel-scale magnetic programming 3D printer

Supplementary Video 2.

Dolphin-like 1D strip magnetic robot wavers underwater

Supplementary Video 3.

Battle-like 1D strip magnetic robot rolling

Supplementary Video 4.

Caterpillar-like 1D strip robot forward and backward crawling

Supplementary Video 5.

2D claw-membrane magnetic robot with multi-mode motions in stomach

Supplementary Video 6.

2D claw-membrane magnetic robot swimming

Supplementary Video 7.

3D spiral capsule robot moves in living rabbit under DSA

Supplementary Video 8.

3D spiral capsule robot moves in 8-shape route

Supplementary Video 9.

3D spiral capsule robot moves in M-shape route

Supplementary Video 10.

Intestine-inspired peristaltic motion

Supplementary Video 11.

Wave-like peristaltic pump motion

**Supplementary Reference**

1. Cao, Q., Han, X., and Li, L. Configurations and control of magnetic fields for manipulating magnetic particles in microfluidic applications: magnet systems and manipulation mechanisms. *Lab Chip* 2014;15(14).

2. Xiao-fan, G., Yong, Y., and Xiao-jing, Z. Analytic expression of magnetic field distribution of rectangular permanent magnets. *Applied Mathematics and Mechanics* 2004;3(25), 297-306.

3. Kim, Y., Yuk, H., Zhao, R., Chester, S.A., and Zhao, X. Printing ferromagnetic domains for untethered fast-transforming soft materials. *Nature* 2018;7709(558), 274-279.

4. Kokkinis, D., Schaffner, M., and Studart, A.R. Multimaterial magnetically assisted 3D printing of composite materials. *Nat. Commun.* 2015;1(6).

5. Liu, W.C., Chou, V.H.Y., Behera, R.P., and Le Ferrand, H. Magnetically assisted drop-on-demand 3D printing of microstructured multimaterial composites. *Nat. Commun.* 2022;1(13).

6. Martin, J.J., Fiore, B.E., and Erb, R.M. Designing bioinspired composite reinforcement architectures via 3D magnetic printing. *Nat. Commun.* 2015;1(6).

7. Kricke, J.L., Yusnila Khairani, I., Beele, B.B.J., Shkodich, N., Farle, M., Slabon, A., Doñate-Buendía, C., and Gökce, B. 4D printing of magneto-responsive polymer structures by masked stereolithography for miniaturised actuators. *Virtual and Physical Prototyping* 2023;1(18).

8. Lantean, S., Roppolo, I., Sangermano, M., Hayoun, M., Dammak, H., Barrera, G., Tiberto, P., Pirri, C.F., Bodelot, L., and Rizza, G. Magnetoresponsive Devices with Programmable Behavior Using a Customized Commercial Stereolithographic 3D Printer. *Adv. Mater. Technol.* 2022;11(7).

9. Xu, T., Zhang, J., Salehizadeh, M., Onaizah, O., and Diller, E. Millimeter-scale flexible robots with programmable three-dimensional magnetization and motions. *Sci.Robot.* 2019;29(4).

10. Alapan, Y., Karacakol, A.C., Guzelhan, S.N., Isik, I., and Sitti, M. Reprogrammable shape morphing of magnetic soft machines. *Sci. Adv.* 2020;38(6).
